# Functional characterization and expression analysis of rice δ^1^-pyrroline-5-carboxylate dehydrogenase provide new insight into the regulation of proline and arginine catabolism

**DOI:** 10.3389/fpls.2015.00591

**Published:** 2015-08-05

**Authors:** Giuseppe Forlani, Michele Bertazzini, Marco Zarattini, Dietmar Funck

**Affiliations:** ^1^Department of Life Science and Biotechnology, University of FerraraFerrara, Italy; ^2^Biology Section, Department of Plant Physiology and Biochemistry, University of KonstanzKonstanz, Germany

**Keywords:** proline and arginine catabolism, enzyme properties, cation and anion effects, gene expression, plant response to stress conditions

## Abstract

While intracellular proline accumulation in response to various stress conditions has been investigated in great detail, the biochemistry and physiological relevance of proline degradation in plants is much less understood. Moreover, the second and last step in proline catabolism, the oxidation of δ^1^-pyrroline-5-carboxylic acid (P5C) to glutamate, is shared with arginine catabolism. Little information is available to date concerning the regulatory mechanisms coordinating these two pathways. Expression of the gene coding for P5C dehydrogenase was analyzed in rice by real-time PCR either following the exogenous supply of amino acids of the glutamate family, or under hyperosmotic stress conditions. The rice enzyme was heterologously expressed in *E. coli*, and the affinity-purified protein was thoroughly characterized with respect to structural and functional properties. A tetrameric oligomerization state was observed in size exclusion chromatography, which suggests a structure of the plant enzyme different from that shown for the bacterial P5C dehydrogenases structurally characterized to date. Kinetic analysis accounted for a preferential use of NAD^+^ as the electron acceptor. Cations were found to modulate enzyme activity, whereas anion effects were negligible. Several metal ions were inhibitory in the micromolar range. Interestingly, arginine also inhibited the enzyme at higher concentrations, with a mechanism of uncompetitive type with respect to P5C. This implies that millimolar levels of arginine would increase the affinity of P5C dehydrogenase toward its specific substrate. Results are discussed in view of the involvement of the enzyme in either proline or arginine catabolism.

## Introduction

Intracellular accumulation of high proline levels as a stress protectant has long been reported in many plant species in response to a wide array of abiotic stress conditions (Verbruggen and Hermans, 2008), ranging from drought, excess salt, and cold (Hayat et al., 2012) to the treatment with heavy metals (Sharma and Dietz, 2006). Besides acting as a compatible osmolyte to counteract water withdrawal from the cytoplasm caused by either hyperosmotic or freezing stress, proline has been reported to stabilize proteins, DNA and membranes, to reduce lipid peroxidation in plants subjected to stress (Kavi Kishor and Sreenivasulu, 2014) and to act as a radical scavenger (Signorelli et al., 2014). On the other hand, high concentrations of proline in the cell increase in turn reactive oxygen species (ROS) formation in the mitochondrion through the activity of a proline dehydrogenase [EC 1.4.3] that is believed to transfer electrons directly to the respiratory chain (Liang et al., 2013). The need to balance ROS production and scavenging to maintain optimal signaling levels for reinstating metabolic homeostasis during stress situations has been well-established (Türkan and Demiral, 2009). The product of proline oxidation, δ^1^-pyrroline-5-carboxylic acid (P5C), is non-enzymatically linearized to glutamate semialdehyde (GSA) and further oxidized by a P5C dehydrogenase [EC 1.5.1.12], yielding glutamate (Forlani et al., 1997a). During the recovery from stress, this short catabolic pathway provides energy, reducing power and precursors for nucleotide synthesis to allow the resumption of cell division (Hare and Cress, 1997).

**Figure 1:**
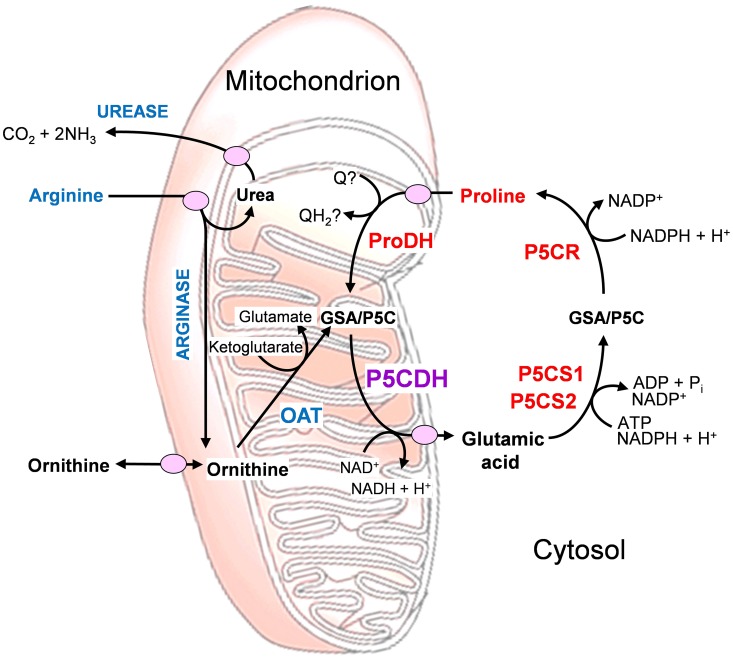
**Inter-related metabolic pathways for arginine and proline catabolism**. The two amino acids are transported into the mitochondrion, where their catabolic routes converge to P5C formation. Abbreviations: GSA, glutamate semialdehyde; OAT, ornithine-δ-aminotransferase; P5C, δ^1^-pyrroline-5-carboxylate; P5CDH, P5C dehydrogenase; P5CR, P5C reductase; P5CS, P5C synthetase; ProDH, proline dehydrogenase; Q, ubiquinone.

In enterobacteria, a proline oxidase associated with the cytoplasmic membrane was shown to possess both proline dehydrogenase and P5C dehydrogenase activity, allowing substrate channeling of P5C between the two active sites of the bifunctional enzyme (Arentson et al., 2012). For all other species tested so far, these activities depended on two distinct proteins. This discrepancy could reflect differences in arginine catabolism, which takes place via ornithine and P5C in most eukaryotes, while some bacteria, namely *E. coli*, use predominantly the arginine succinyltransferase pathway, which does not use P5C as an intermediate (Schneider et al., 1998; Winter et al., 2015). The presence of a distinct P5C dehydrogenase may enable better, simultaneous regulation of the two convergent pathways of arginine and proline catabolism (Figure 1).

Measurements of proline-dependent oxygen uptake of mitochondria isolated from water-stressed barley and maize plants suggested that the modulation of proline channeling into the respiratory chain could play a role in stress-induced proline accumulation (Stewart et al., 1977; Sells and Koeppe, 1981). When proline dehydrogenase and P5C dehydrogenase activities were measured separately in mitochondria from osmotically-stressed maize seedlings, the former was found to be strongly reduced, whereas P5C dehydrogenase activity was unaffected. The occurrence of stress-driven regulating mechanism(s) was thus inferred exclusively for the first enzyme in proline catabolism (Rayapati and Stewart, 1991). More recently, molecular data confirmed a strong induction of proline dehydrogenase following release from hyperosmotic stress or the treatment with exogenous proline (Funck et al., 2010; Szabados and Savouré, 2010), and showed its connections within the mitochondrial metabolism in plants (Schertl et al., 2014).

Despite the protective role under stress, exogenously supplied proline was found to exert phytotoxic effects and trigger the activation of programmed cell death (PCD) (Deuschle et al., 2004). An early induction of the gene for P5C dehydrogenase was shown in several crops infected by virulent fungal strains (Mitchell et al., 2006). Moreover, induction of proline oxidation was reported in *Arabidopsis thaliana* during incompatible plant-pathogen interactions (Cecchini et al., 2011). Therefore the possibility exists that proline metabolism is also involved in the process leading to PCD during the hypersensitive defense reaction (Senthil-Kumar and Mysore, 2012). However, it is still unclear which may be the active molecule, whether proline, P5C or ROS produced during proline catabolism. Interestingly, the early activation of proline dehydrogenase during pathogen attack was accompanied by an increase in P5C reductase but not in P5C dehydrogenase transcripts, apparently with few changes occurring in proline and P5C levels (Cecchini et al., 2011). Therefore, some authors postulated that P5C might trespass the mitochondrial membrane, and that a P5C/proline cycle would occur as a consequence, leading to reactive oxygen species production (Miller et al., 2009). This cycle would be mediated by the activities of proline dehydrogenase in the mitochondrion and P5C reductase in the cytosol (Figure 1). Indeed, the analysis of wild-type *A*. *thaliana* plants and *p5cdh* mutants showed that the absence of P5C dehydrogenase does not reduce ROS production, cell death, or pathogen resistance, and suggested that the enzyme does not act synergistically with proline dehydrogenase in the potentiation of such defense responses (Monteoliva et al., 2014). In any case, the exact mechanisms underlying the role of proline metabolism under biotic stress conditions still await full elucidation.

Interestingly, the gene coding for P5C dehydrogenase in *A. thaliana* was found regulated by endogenous siRNAs co-ordinating the cell response to salt stress conditions. *AtP5CDH* was shown to partially overlap on opposite strands with *SRO5*, a gene of unknown function. The former seems constitutively expressed, whereas the latter is salt-stress inducible. When both genes are transcribed, a 24-nt *SRO5*-*P5CDH* siRNA is formed, promoting the degradation of *P5CDH* transcripts (Borsani et al., 2005). This mechanism could be functional to fuel the P5C/Pro cycle, thereby increasing ROS production (Miller et al., 2009). However, this would apparently conflict with the proposed role for SRO5, which is believed to counteract ROS generation (Borsani et al., 2005).

Moreover, when the mRNA levels of the aldehyde dehydrogenase gene superfamily were measured in rice (*Oryza sativa* L.) seedlings under different experimental conditions, the expression of some genes was found up-regulated by drought and salinity and the exogenous application of abscisic acid (ABA), indicating that the products of these genes are potentially involved in rice osmotic stress adaptation. Quite surprisingly, the gene encoding P5C dehydrogenase (*OsP5CDH*, also referred to as *OsALDH12*, locus name *OsJ_19352*) was unaffected by salinity stress, and induced (rather than repressed) by drought and ABA treatments (Gao and Han, 2009). However, in that study neither the corresponding intracellular levels of proline nor a time course of gene expression during the stress were shown.

In addition to the uncertainties on the modulation at the transcriptional level in response to environmental signals, very little is known about the occurrence of post-translational regulation mechanisms for P5C dehydrogenase. In early studies, maximal activity was detected in crude mitochondrial preparations after detergent treatment. Biochemical characterization pointed out a clear-cut preference for NAD^+^ as the electron acceptor, and *K*_M_ values in the range of 10^−4^ to 10^−3^ M for both P5C and NAD^+^ (Stewart and Lai, 1974; Boggess et al., 1975). Biochemical analyses supported the occurrence of two distinct enzyme forms, each one specifically involved in either proline or arginine oxidation in maize and wild tobacco (Elthon and Stewart, 1981; Forlani et al., 1997b). However, in most diploid genomes analyzed so far, including *A. thaliana* and *O. sativa*, only a single *P5CDH* gene seems to exist (Deuschle et al., 2001; Ayliffe et al., 2005). To date, plant P5C dehydrogenase has been purified only from a *Solanum tuberosum* cell culture (Forlani et al., 1997a). The characterization of the potato enzyme showed that NADP^+^ can also serve as electron acceptor, and that NaCl inhibits enzyme activity in a concentration-dependent manner. A subsequent analysis of protein levels in plant tissues and in response to salt stress and exogenous proline suggested a developmental, but not an environmental control of P5C dehydrogenase expression. This notwithstanding, protein levels showed an overall positive correlation with free proline concentration inside the cell (Forlani et al., 2000).

In the frame of a research project for integrated genetic and genomic approaches for new Italian rice breeding strategies, we aim at a better understanding of the biochemical mechanisms underlying salt tolerance in rice. To achieve this goal, the regulative switches responsible for the intracellular accumulation of proline under hyperosmotic stress conditions need to be identified. Here, we report a thorough characterization of rice P5C dehydrogenase, affinity-purified after heterologous expression in *E. coli*. Activity was inhibited by cations and arginine in the millimolar range, whereas some heavy metals were effective in the micromolar range. In cultured rice cells, mRNA levels were measured by real-time PCR, showing a constitutive expression of *OsP5CDH*, as well as slightly enhanced transcript levels in response to PEG treatments.

## Materials and methods

### Plant materials and growth conditions

Suspension cultured cells of rice (*Oryza sativa* L. ssp. *japonica, cultivars* Vialone nano and Loto) were grown in MS medium (Murashige and Skoog, 1962) supplemented with 30 g L^−1^ sucrose and 2 mg L^−1^ 2,4-dichlorophenoxyacetic acid, as previously described (Rożkowicz et al., 2003). Cultures were maintained in continuous balanced growth by subculturing 12 mL-aliquots to 50 mL of fresh medium in 250 mL Erlenmeyer flasks every 7 d.

Hyperosmotic stress conditions were obtained either by adding to the culture medium a suitable volume of a 5 M solution of NaCl in water, or by resuspending cells in MS medium containing PEG 6000 at concentrations ranging from 15 to 25% (w/v). Exogenous supply of amino acids was performed through the addition of proper volumes of 1 M solutions, sterilized by filtration (0.22 μm). To avoid pH-dependent non-specific effects, arginine was used as its hydrochloride. Abscisic acid was prepared as a 50 mM solution in pure ethanol. All treatments were applied directly at subculturing, and were carried out in triplicate. Cell viability was measured by the Evans' blue uptake assay, as described (Rożkowicz et al., 2003).

### Reverse transcriptase real-time PCR analysis

Total RNA was extracted from 50 mg of plant material by using the Plant RNA Isolation kit (Agilent Technologies), according to the manufacturer's instructions. Following spectrophotometric quantification and assessment of RNA integrity, RNA (1 μg) was incubated at 30°C for 30 min with 5 U of amplification grade DNase I (Sigma AMPD1), treated for 10 min at 70°C to denature both the DNase I and the RNA, chilled on ice and immediately used for single strand cDNA synthesis by reverse transcription in a 20 μL reaction mixture using the iScript cDNA Synthesis Kit (Bio-Rad). First strand cDNA was used as template for PCR amplifications using specific primers for target and housekeeping genes (Table 1). For semi-quantitative PCR analysis, the amplification was carried out in a Mastercycler Personal thermal cycler (Eppendorf) in a 20 μL reaction mixture containing 10 μL iQTMSupermix (Bio-Rad), 20 pmol forward and reverse primers, and 50 ng cDNA. Cycling conditions consisted of an initial 5 min at 95°C, followed by 60 s denaturing at 94°C, 30 s annealing at 61°C, and 60 s elongation at 72°C repeated for a given number of cycles, and with 5 min final extension at 72°C. The exponential phase of amplification was determined by analyzing gel (1.5% agarose) electrophoresis pattern of the PCR products generated with different numbers of cycles. qPCR was carried out with 25 ng cDNA in a volume of 15 μL on a CFX96 Real-Time PCR Detection System (Bio-Rad) using SsoAdvanced™ Universal SYBR® Green Supermix (Bio-Rad) with the following thermal profile: enzyme activation at 98°C for 30 s, followed by 40 cycles of denaturation for 5 s at 95°C and annealing and extension for 15 s at 61°C. Melting curve analysis was performed after cycle completion to validate amplicon identity. The expression levels of both housekeeping genes were used to obtain the relative expression of each target gene by the ΔΔ_Ct_ method (Pfaffl, 2001) using the Gene Expression Module of CFX Manager software™ (Version 3.1, Bio-Rad). For each treatment, three biological replications were performed, and two technical replicates were run for each sample in qPCR analysis.

**Table 1:** **Specific primers used for RT-PCR and qRT-PCR**.

**Gene**	**Primers**	**cDNA product size**	**Genomic DNA product size**
*P5C synthetase 1* (protein ID Q941T1)	fwd CGCAGGATCAATTCGTGAAATCGCA rev GCAATCTGTACCAAGGCATCAGGA	162	291
*P5C synthetase 2* (protein ID O04226)	fwd GATTGGGTGCTGAGGTTGGCATAA rev CGACATCCTTGTCACCATTCACCA	132	–
*P5C reductase* (protein ID Q8GT01)	fwd ATCGTAAGGCAGGTACTGGTTGAG rev AGCTGAACGGGTGTTTGGCATT	150	377
*Ornithine*-δ*-aminotransferase* (protein ID Q10G56)	fwd GAGGGGTTTGCTTAATGCAGTGGA rev CAGCGGAGGCGCTAATCTGATTAT	142	142
*Proline dehydrogenase* (protein ID Q336U3)	fwd ACGGTCCAGTGGAGCAGATCAT rev AGCATTGCAGCCTTGAACCT	133	261
*P5C dehydrogenase* (protein ID B9FLB9)	fwd ATGCTTGCAGTGGTCAGAAGTG rev TGGGCCAATGGTCAAGTCTTCA	134	487
*Actin-1* (protein ID Q10DV7)	fwd CTGCGGGTATCCATGAGACT rev GCAATGCCAGGGAACATAGT	118	118
*Elongation factor (EF) 1*α (protein ID O64937)	fwd TCTCTGGGTTTGAGGGTGACAACA rev TTGATCTGGTCAAGAGCCTCAAGC	94	94

*Sequences were designed using the Primer 3 software (http://bioinfo.ut.ee/primer3-0.4.0/)*.

### Amino acid extraction and analysis

Cells were harvested by vacuum filtration on nylon filters (50 μm mesh), weighed and ground in a mortar with 2 mL g^−1^ of a 3% (w/v) solution of 5-sulphosalicylic acid. Following centrifugation for 10 min at 12,000 *g*, the supernatant was mixed with the same volume of *o*-phthaldialdehyde solution [0.5 M in 0.5 M sodium borate buffer, pH 10.0, containing 0.5 M β-mercaptoethanol, and 10% (v/v) methanol]. After exactly 60 s, 20 μL of derivatized samples were injected onto a 4.6 × 250 mm Zorbax ODS column (Agilent Technologies) equilibrated with 59% solvent A [50 mM sodium phosphate-50 mM sodium acetate buffer, pH 7.5, containing 2% (v/v) of both methanol and tetrahydrofuran] and 41% solvent B (65% methanol). Elution proceeded at a flow rate of 60 mL h^−1^ using a computer-controlled (Data System 450; Kontron, Munich, Germany) complex gradient from 41 to 100% solvent B, as described (Forlani et al., 2014), monitoring the eluate at 340 nm. Proline and total amino acid content were quantified by a modification of the acid ninhydrin method (Williams and Frank, 1975). Data were expressed on a fresh weight basis.

### Cloning of *OsP5CDH* and heterologous expression in *E. coli*

The coding sequence of *O. sativa* P5C dehydrogenase was amplified by PCR from cDNA clone *J033091O16* (Rice Genome Resource Center, National Institute of Agrobiological Sciences DNA Bank, Japan) with the primers P5CDH-fwd (caccTCAGGtCCaGCCGCACTC) and P5CDH-rev (GTCAAGGCTAGATTAGCTTGTAGC), and inserted into the expression vector pET151 by directional TOPO cloning (Life Technologies, Carlsbad, CA, USA), yielding the vector pET151-*Os*P5CDH. The same procedure carried out with the primers ntP5CDH-fwd (caccATGAGCCTCATCCTTTCCCG) and P5CDH-rev yielded the vector pET151-nt*Os*P5CDH coding the non-truncated form of the protein bearing its mitochondrial transit peptide at the amino terminus (Supplementary Figure 1).

For heterologous expression, *E. coli* BL21(DE3) pLysS cells (Invitrogen) were made competent by the calcium chloride method, transformed with either vector and selected on ampicillin-containing LB plates. After inducing the expression of P5C dehydrogenase by 1 mM isopropyl-D-thiogalacto-pyranoside (IPTG) at 24°C, the cells were lysed in a mortar with 2 g g^−1^ alumina and resuspended in 20 mL g^−1^ extraction buffer (50 mM Na phosphate buffer, pH 7.5, containing 200 mM NaCl, 0.5 mM DTT, and 20 mM imidazole). The His-tagged protein was purified from clarified extracts by affinity chromatography with a His-Select™ Nickel Affinity Gel column (1.5 mL bed volume, Sigma H7788). Stepwise elution was achieved by increasing concentrations of imidazole in extraction buffer. For activity assays, the purified enzyme was diluted 1:100 with water, and a proper aliquot (2 to 5 μL) was added to the assay mixture. To remove the His-tag, aliquots (50 μg) of the preparation were treated with 0.5 μg of tobacco etch virus (TEV) protease (Sigma T4455), according to the cleavage protocol provided by the manufacturer.

### P5C dehydrogenase assay

Enzyme activity was measured at 35°C as the P5C-dependent reduction of NAD(P)^+^. The assay mixture contained 50 mM Hepes-KOH buffer, pH 7.5, 10 mM NAD^+^ or 20 mM NADP^+^, and 1 mM l-P5C in a final volume of 0.2 mL. A limiting amount of enzyme (from 0.2 to 1 μg of the purified protein) was added to the pre-warmed mixture and the increase in absorbance was determined at 340 nm for up to 30 min at 1-min intervals against blanks from which P5C had been omitted. Activity was determined from the initial linear rate, based on the assumption of an extinction coefficient for NAD(P)H of 6220 M^−1^ cm^−1^. Linear regression analysis was computed by using Prism 6 (version 6.03, GraphPad Software, Inc., USA). dl-P5C was synthesized by the periodate oxidation of δ-allo-hydroxylysine (Sigma H0377) and purified by cation-exchange chromatography, as described previously (Forlani et al., 1997a). l-P5C was assumed to represent 50% of the added P5C concentration, and d-P5C was neglected because it has been shown to be biologically inactive in the assay (Williams and Frank, 1975). Therefore, throughout this study, P5C refers to l-P5C only. The protein concentration was determined by the method of Bradford (1976), using bovine serum albumin (BSA) as the standard. For the purified protein, direct absorbance at 280 nm was used instead, and the concentration was calculated on the basis of a deduced molar extinction coefficient for rice P5C dehydrogenase of 67,630 M^−1^ cm^−1^ (http://web.expasy.org/protparam).

### Kinetic analyses

To evaluate substrate affinity, unvariable substrates were fixed at the same levels as in the standard assay. The concentration of l-P5C ranged from 100 to 1000 μM; NAD^+^ or NADP^+^ were added in the range from 0.5 to 10 mM and from 1 to 20 mM, respectively. To evaluate the mechanism of the inhibition brought about by arginine, NAD^+^ and P5C concentration ranged from 1 to 7 mM and from 100 to 700 μM, respectively. When evaluating the effect of ions, l-P5C concentration was reduced to 250 μM to minimize the carry-over of chloride anions, and Hepes buffer was used at 12.5 mM to reduce the content of potassium cations; in this case, pH was adjusted with NH_3_, which at concentrations below 100 mM had no effect on enzyme activity. All assays were performed in triplicate. *K*_M_ and V_max_ values, as well as the concentrations causing 50% inhibition (IC_50_) of P5C dehydrogenase activity, *K*_I_-values and their confidence intervals were estimated by non-linear regression analysis using Prism 6. Activation energy was obtained by the replotting (Arrhenius plot) of data from two independent experiments in which the catalytic rate was measured as a function of temperature in the range 25 to 42.5°C, at 2.5°C-intervals. Thermostability of the enzyme was determined by incubating the purified protein for various intervals of time at 40 to 50°C, followed by determination of the remaining activity under standard assay conditions. Catalytic constants were calculated from V_max_ values taking into account a homotetrameric composition of the native holoenzyme, with each monomer having a molecular mass of 57,670 Da.

### Determination of isoelectric point, native, and denatured molecular mass

Discontinuous SDS-polyacrylamide gel electrophoresis was performed at 20°C by the method of Laemmli with a 4% stacking and a 8% separating gel, using a Minigel system (BioRad). Samples were denatured by boiling 5 min in 62 mM Tris-HCl buffer (pH 6.8), containing 2% (w/v) SDS, 10% (v/v) glycerol, and 5% (v/v) β-mercaptoethanol. Proteins were visualized after staining with 0.1% Coomassie brilliant blue R-250.

Gel permeation chromatography was performed by injecting 100-μL aliquots of the purified protein onto a Superose 12 HR 10/30 (Pharmacia) column that had been equilibrated with 50 mM Tris-HCl buffer, pH 7.75, containing 250 mM NaCl. Elution proceeded at the constant flow of 0.5 mL min^−1^, for the collection of 0.5-mL fractions, while monitoring the eluate at 280 nm (HPLC Detector 432, Kontron). Molecular weight markers for column calibration (Pharmacia) were bovine thyroid thyroglobulin (669 kDa), horse spleen ferritin (440 kDa), bovine liver catalase (232 kDa), rabbit muscle aldolase (158 kDa), and BSA (67 and 268 kDa). Three runs were carried out for each marker, and six runs for the purified protein.

Isoelectric focusing was performed as described previously (Forlani et al., 1997a), with ampholytes within the pH 3.5–10 range (Pharmacia). pI markers (Sigma) were bovine milk β-lactoglobulin A (pI 5.1), bovine erythrocyte carbonic anhydrase II (5.4 and 5.9), and bovine erythrocyte carbonic anhydrase I (6.6). After the run, individual tracks were cut from the gel and either sliced in 5-mm segments for the determination of pH, or stained for protein as above.

## Results

### Expression of rice *P5CDH* under hyperosmotic stress conditions, and in response to the exogenous supply of metabolically related amino acids

**Figure 2:**
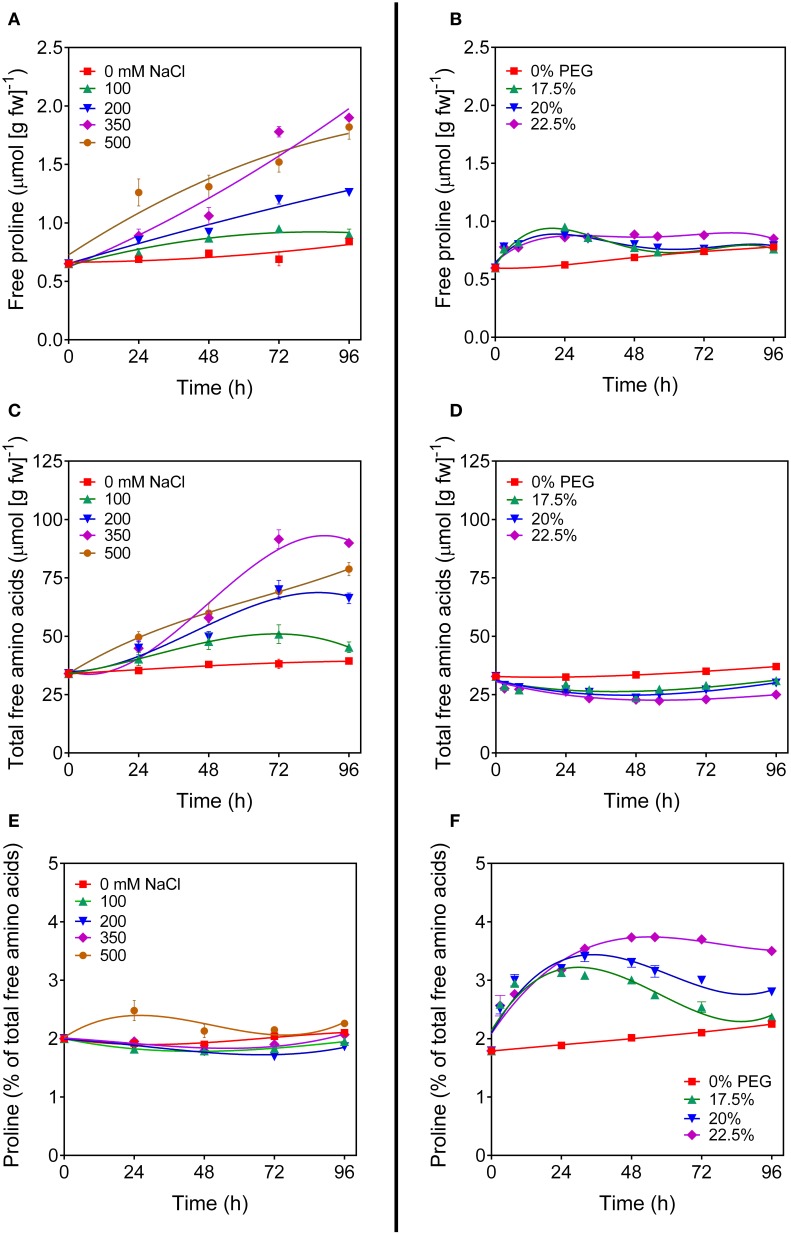
**Proline levels in salt- and PEG-treated rice cells**. Suspension cultured cells of rice (*cv* Vialone nano) were treated with different, growth inhibitory concentrations of either NaCl **(A,C,E)** or PEG 6000 **(B,D,F)**. At increasing time after the treatments, free proline levels **(A,B)** and total amino acid concentrations **(C,D)** were determined on a fresh weight basis. Proline concentration was also expressed as percent of total amino acids **(E,F)**. All treatments were carried out in triplicate, and means ± SE are depicted. Very similar results were obtained also with cell cultures of the *cv* Loto.

To investigate the regulation of *OsP5CDH* during stress-induced proline accumulation, rice suspension cultured cells were treated with increasing concentrations of either NaCl or polyethylene glycol (PEG) to induce salt or osmotic stress conditions, respectively. Cell growth was progressively reduced by both stress treatments, but not completely suppressed (not shown). As expected, free proline content in salt-stressed cells increased with time in a dose-responsive manner (Figure 2A). However, also total amino acid content showed a similar pattern (Figure 2C), and if proline content was expressed as percentage of total amino acids, only minor variations, if any, were evident in response to salt treatment (Figure 2E). A remarkably different picture was obtained with PEG. In this case only a slight but reproducible increase of intracellular proline levels was found soon after the exposure to osmotic stress conditions, which at low PEG doses came back to control levels after a couple of days (Figure 2B). The concentration of free amino acids did not increase, but showed on the contrary a slight decrease (Figure 2D). Because the treatment caused a 20 to 35%-loss of cell viability (data not presented), such a decrease most likely depended on the presence of a significant amount of dead cells that influences the amino acid content, as expressed on a fresh weight basis. Interestingly, when proline levels were expressed as percentage of total amino acids, a significant increase was evident that was proportional to the severity of the stress. At 22.5% PEG, this increase was maintained over the entire culture cycle (Figure 2F). The effects of higher PEG concentrations were not tested because of the resulting undesirable consequence on cell viability. Because the plant response to hyperosmotic stress conditions is at least in part mediated by abscisic acid, similar experiments were performed also by treating cells with 50 μM ABA, either alone or in combination with PEG. Results were identical to those obtained for untreated cells or with cell treated with PEG alone, respectively (data not shown). To rule out the possibility that these results could be cultivar-specific, or depend on some mutation that may have occurred with time at the undifferentiated tissue level, where mutations that are lethal *in planta* can be maintained within the cell population, the experiments were carried out with independent cell cultures of two *japonica* rice cultivars (Loto and Vialone nano). Virtually overlapping patterns of amino acid profile changes were found in both cell cultures (data not shown).

**Figure 3:**
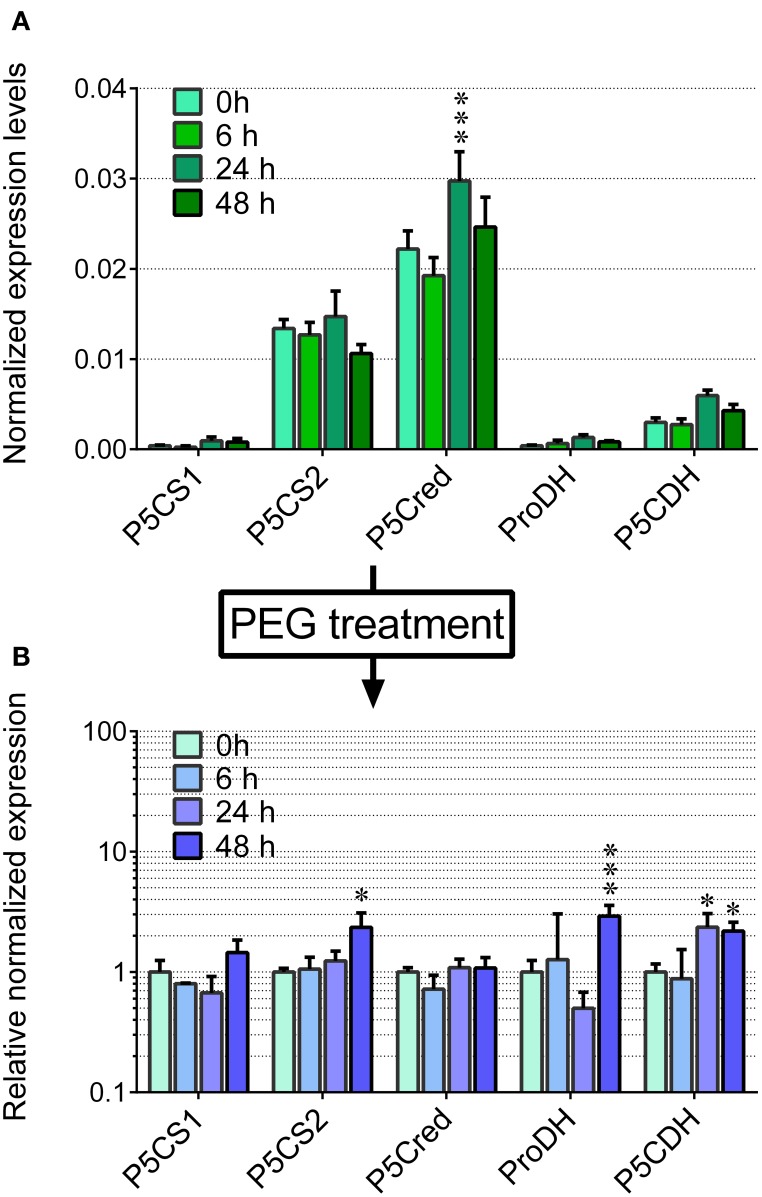
**Transcript levels of the genes involved in proline synthesis from glutamate and its re-oxidation in PEG-treated rice cells**. The expression levels were quantified in suspension cultured cells of rice (*cv* Vialone nano) by real-time PCR following reverse transcription. Levels were normalized with respect to *Actin 1* (Os03t0718100-01) cDNA **(A)**. The same experiment was performed with cells treated with 22.5% PEG 6000, harvested at increasing time after the treatment. Expression levels were in this case normalized with respect to those in untreated controls **(B)**. All treatments were carried out in triplicate, and means ± SE are reported. Results were subjected to analysis of variance, taking into account the Bonferroni correction for multiple testing; ^*^, ^***^: significantly different at the 10% and 1% level, respectively, with respect to time 0 **(A)** or untreated controls **(B)**.

Based on these pieces of evidence, the effect of the treatment with 22.5% PEG on the expression level of the genes coding for the enzymes involved in proline synthesis and catabolism was investigated. In unstressed cells, *P5C synthetase 2* and *P5C reductase* mRNAs were present at significantly higher levels than that of *OsP5CDH*, whereas *P5C synthetase 1* and *Proline dehydrogenase* transcripts were almost undetectable (Figure 3A). In PEG-stressed cells, despite the rapid and relevant increase of free proline, mRNA levels of *P5C reductase* did not change at all, and only minor variations were found for the two *P5C synthetases*, with a two-fold increase 48 h after the start of the treatment. Concerning the mRNA for *P5C dehydrogenase*, a two-fold increase was also evident, and the same trend was shared by the transcript of *Proline dehydrogenase* (Figure 3B).

**Figure 4:**
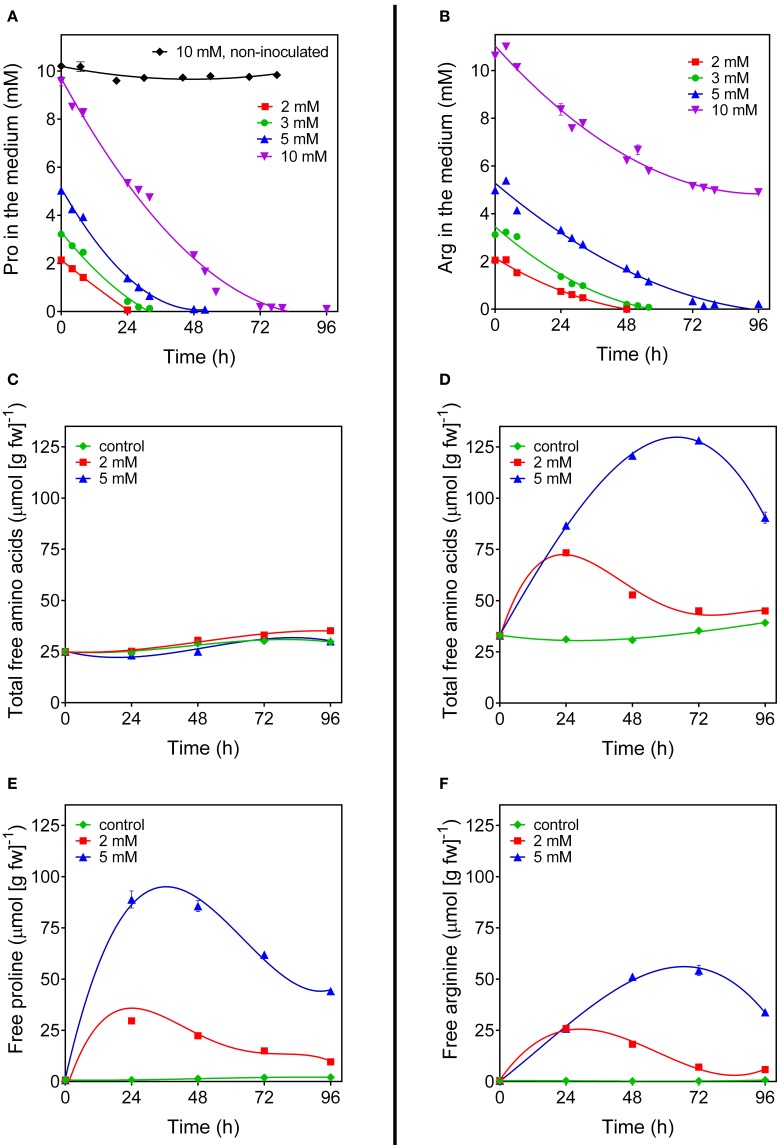
**Arginine and proline utilization in amino acid-fed rice cells**. Suspension cultured cells of rice (*cv* Loto) were supplied with different concentrations of either proline **(A,C,E)** or arginine **(B,D,F)**. At increasing time after the addition of the exogenous amino acid, its residual level in the medium was determined **(A,B)**. Cells from the same samples were harvested and washed thoroughly before intracellular levels of total amino acid **(C,D)**, free proline **(E)** and free arginine **(F)** were determined on a fresh weight basis. All treatments were carried out in triplicate, and means ± SE are reported. Virtually overlapping patterns were obtained with cell cultures of the *cv* Vialone nano.

**Figure 5:**
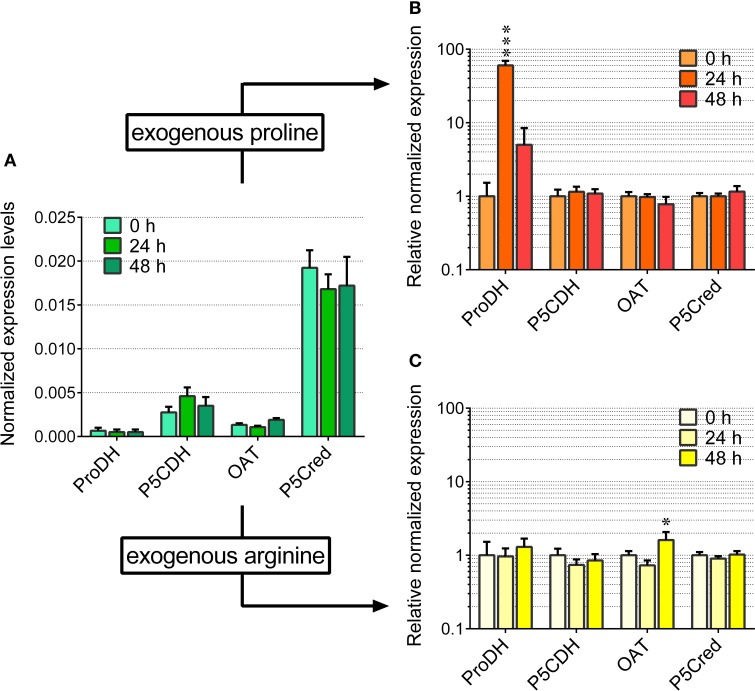
**Transcript levels of the genes involved in proline and arginine catabolism in arginine- and proline-fed rice cells**. The expression of the genes involved in proline and ornithine oxidation to glutamate was quantified in suspension cultured cells of rice (*cv* Loto) by real-time PCR following reverse transcription. Levels were normalized with respect to *Actin 1* cDNA **(A)**. The same experiment was performed with cells treated with either 2 mM proline **(B)** or 2 mM arginine **(C)**, harvested at increasing time after the addition of the exogenous amino acid. Expression levels were in this case normalized with respect to those in untreated controls. All treatments were carried out in triplicate, and means ± SE are reported. Results were subjected to analysis of variance, taking into account the Bonferroni correction for multiple testing; ^*^, ^***^: significantly different at the 10% and 1% level, respectively, from time 0 **(A)** or untreated controls **(B,C)**.

Even under the best conditions found, the increase of proline concentration in the cells appeared too slight to allow the investigation of its utilization during a subsequent relief from the stress. As an alternative, the fate of exogenously-supplied proline was studied. Rice cells were treated with millimolar concentrations of proline. The addition did not significantly affect cell viability up to 4 days after the treatment (results not shown). Under the experimental conditions adopted, exogenous proline was rapidly and actively taken up by the cells, whereas no significant decrease of proline concentration was found in the absence of cells during the time of the experiment (Figure 4A). Intracellular proline levels reached their maximum 24 h after addition of proline to the medium (Figure 4E). Thereafter, intracellular concentrations slowly came back to control levels. On the contrary, no variations were found with respect to total free amino acid content (Figure 4C). Because the absolute amount of proline utilized was higher in cells treated with 5 mM proline than in cells treated with 2 mM proline (Figure 4E), it is likely that proline is not simply used for protein synthesis for growth. Indeed, transcript level analysis of the genes involved in P5C metabolism in proline-fed cells revealed that 24 h after proline supply *Proline dehydrogenase* transcript levels were 5-fold higher than those of *OsP5CDH* and *Ornithine*-δ*-aminotransferase* (*OAT*), the gene encoding for the enzyme catalyzing the conversion of ornithine into P5C. This corresponds to a 60-fold increase of *Proline dehydrogenase* transcripts over basal levels (Figure 5A). At 48 h after proline treatment the *Proline dehydrogenase* transcript came back to control levels. Notwithstanding this, the mRNA levels of *OsP5CDH* were unaffected at both analyzed time points (Figure 5B).

Since P5C dehydrogenase is potentially involved also in arginine catabolism, the same approach was carried out with exogenous arginine. In this case amino acid uptake proceeded more slowly, with significant levels of the amino acid still present in the culture medium 2 days after the treatment with 5 mM arginine (Figure 4B). Consistently, intracellular concentrations of free arginine increased more slowly, and reached lower values (Figure 4F). In this case the increase of total free amino acids exceeded that of arginine alone (Figure 4D vs. Figure 4F), suggesting the rapid conversion of arginine into other amino acids. Strikingly, no significantly higher levels of ornithine, the immediate degradation product of arginine, were found in cell extracts (data not presented), although *OAT* transcript levels did not show remarkable induction, with only a two-fold increase 48 h after arginine feeding (Figure 5C). Again, also transcript levels of *OsP5CDH* remained largely constant, indicating that enhanced conversion of arginine into other amino acids does not require transcriptional up-regulation of either *OAT* or *OsP5CDH*.

### Cloning, heterologous expression in *E. coli* and affinity purification of rice P5C dehydrogenase

**Figure 6:**
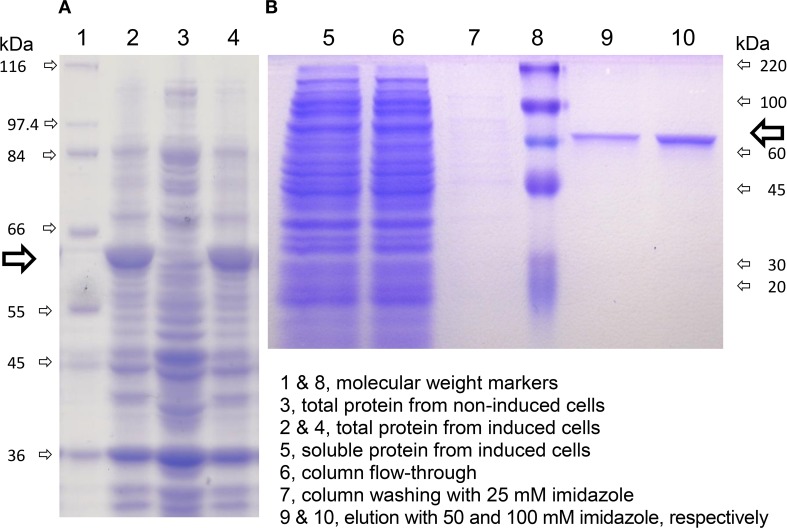
**Expression of rice P5C dehydrogenase in *E. coli* and affinity purification**. A truncated version of rice P5C dehydrogenase lacking the predicted mitochondrial transit peptide (Supplementary Figure 1) was expressed in *E. coli*, strain BL21 DE3 pLysS. Cells were analyzed by SDS-PAGE before and after the induction with 1 mM IPTG **(A)**. Six hours after IPTG addition, cells were harvested and extracted, and the plant protein (⇨) was purified by affinity chromatography on a His-Select™ Nickel Gel column **(B)**. The molecular mass of protein standards is indicated.

The cDNA of the only recognizable gene coding for P5C dehydrogenase in the *japonica* rice genome, *OsP5CDH*, was subcloned into the expression vector pET151. Because the *OsP5CDH* open reading frame codes for a pre-protein including a mitochondrial transit peptide, two constructs were prepared producing the full-length pre-protein or a version truncated by 16 amino acids at the N-terminus, corresponding to the predicted transit peptide (Supplementary Figure 1A). Both constructs yielded strong inducible expression of rice P5C dehydrogenase in *E. coli* (Figure 6A, lanes 2 and 4). However, most of the recombinant protein was sequestered in inclusion bodies, and the corresponding protein band was barely detectable in the soluble protein fraction (Figure 6B, lane 5). As a consequence, following affinity chromatography the final yield was quite low, with an average of about 390 μg protein (g of induced cells)^−1^ for the truncated version, and only 26 μg protein (g of induced cells)^−1^ for the non-truncated form of P5C dehydrogenase. The latter was found catalytically active, with an activity rate corresponding to about 32% of that of the protein without the predicted transit peptide. However, only the truncated protein was fully characterized. Identification of peptic and tryptic peptides by LC-MS/MS analysis confirmed that the purified protein corresponds to the deduced amino acid sequence of the pET151-*OsP5CDH* expression construct (Supplementary Figure 1B).

The presence of the His_6_-tag did not affect the enzymatic activity, since virtually identical results were obtained in all experiments before and after the cleavage of the purified protein with TEV protease. Enzyme preparations were relatively stable at low temperatures; if sterilized by filtration (0.22 μm pore size), more than 70% of activity was retained after 1-month storage at 4°C.

### Functional and structural characterization of rice P5C dehydrogenase

**Figure 7:**
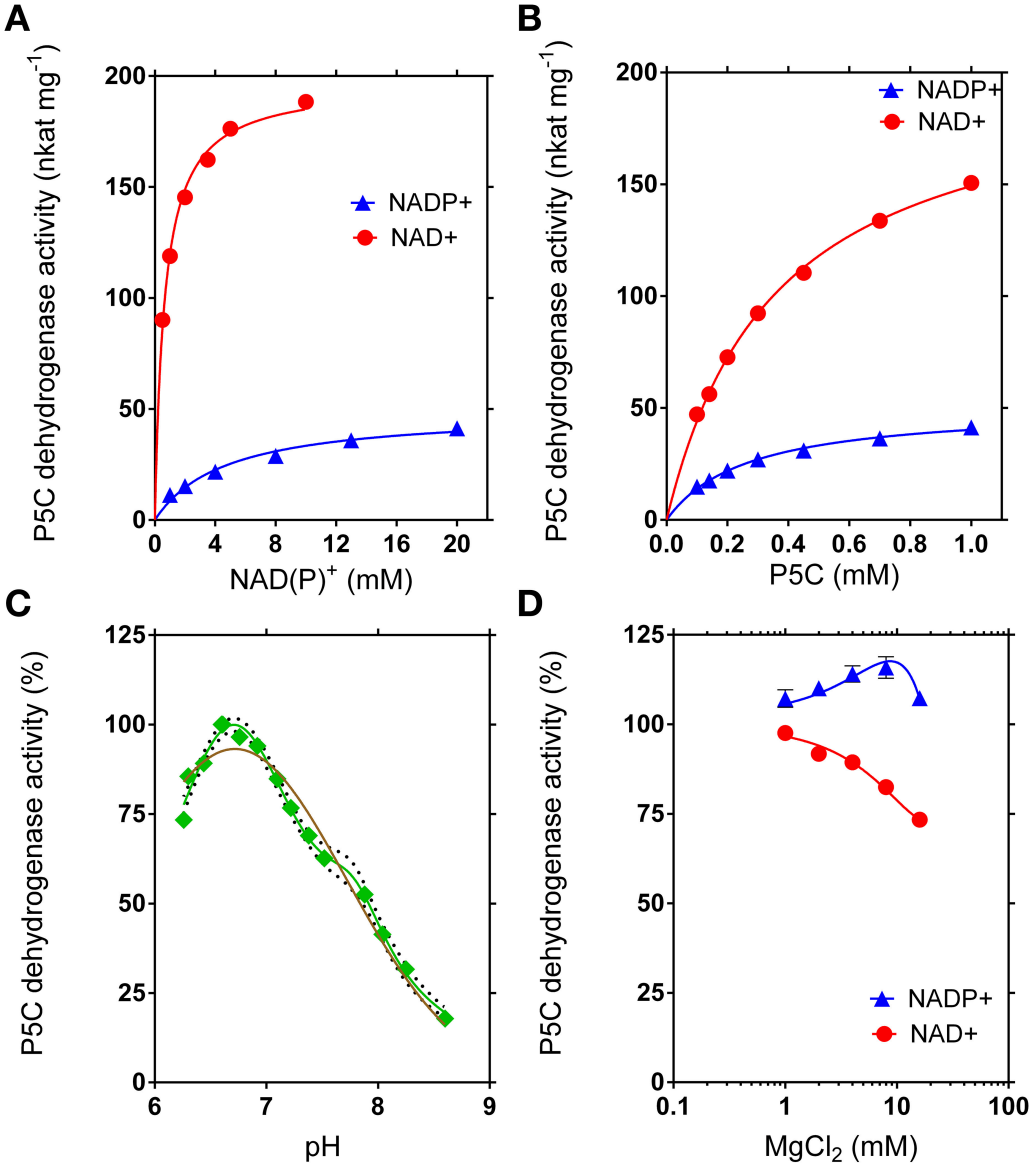
**Kinetic features of rice P5C dehydrogenase**. The activity of purified recombinant rice P5C dehydrogenase (0.5 μg) was measured for up to 30 min at 35°C in the presence of increasing concentrations of NAD(P)^+^ **(A)** or L-P5C **(B)**. Invariable substrates were fixed at the same levels as in the standard assay. Plotting of data from Michaelis–Menten graphs into the Lineweaver–Burk double reciprocal plots allowed the calculation of affinity constants and V_max_ values for the NAD^+^- and the NADP^+^-dependent reaction (Table 2). The pH-dependence of enzyme activity was determined using NAD^+^ as the electron acceptor **(C)**. Better fiting of the experimental results was obtained assuming the sum of two gaussian distributions (green line *r*^2^ = 0.9834) than a single gaussian (brown line; *r*^2^ = 0.9504). The 95%-confidence intervals of the adopted interpolating curve are indicated by a dotted line. The effect of Mg^2+^ ions in the range from 1 to 10 mM was evaluated by adding MgCl_2_ to the standard reaction mixture **(D)**. In all cases, at least three replicates were carried out for each treatment, and mean values ± SE are presented.

The recombinant enzyme was thoroughly characterized with respect to functional and structural properties (Table 2). Interestingly, maximal activity was found at pH 6.72, an unusual feature for an enzyme that is functionally located in the mitochondrial matrix. However, the curve for the pH-dependence of the specific activity was much better fitted as a superimposition of two Gaussian distribution curves with maxima at pH 6.70 and pH 7.79 than by a curve with a single maximum (Figure 7C). An isoelectric point of 5.60 was experimentally determined, in good agreement with that calculated *in silico* (5.84). The inclusion of Mg^2+^ ions into the reaction mixture at millimolar concentrations only slightly influenced enzyme activity, with a mild stimulation when NADP^+^ was used as the electron acceptor, and a slight inhibition if NAD^+^ was the co-factor (Figure 7D).

**Table 2:** **Properties of rice P5C dehydrogenase**.

Denatured molecular mass (by SDS-PAGE)	58.2 ± 1.3 kDa
Native molecular mass (by gel permeation)	259 ± 14 kDa
Isoelectric point	5.60 ± 0.05
pH optimum	6.72
Temperature optimum	46 ± 1°C
Activation energy	54.6 ± 3.7 kjoules mol^−1^
V${}_{\text{max }\left({NAD}^{+}\right)}$	196.9 ± 2.1 nkat (mg protein)^−1^
V${}_{\text{max }\left(P5C,{with\;NAD}^{+}\;as\;the\;co-substrate\right)}$	207.7 ± 3.3 nkat (mg protein)^−1^
V${}_{\text{max }\left({NADP}^{+}\right)}$	49.2 ± 1.7 nkat (mg protein)^−1^
V${}_{\text{max }\left(P5C,{with\;NADP}^{+}\;as\;the\;co-substrate\right)}$	51.0 ± 1.0 nkat (mg protein)^−1^
K${}_{\text{cat }\left({NAD}^{+}\right)}$	12 s^−1^
K${}_{\text{cat }\left({NADP}^{+}\right)}$	3 s^−1^
K_M(app)_ for L-P5C ${}_{\left(\text{NA}{\text{D}}^{+}\right)}$	358 ± 14 μM
K_M(app)_ for L-P5C ${}_{\left(\text{NAD}{\text{P}}^{+}\right)}$	265 ± 13μM
K_M(app)_ for NAD^+^	644 ± 30 μM
K_M(app)_ for NADP^+^	4695 ± 462 μM

#### Rice P5C dehydrogenase can use either NAD^+^ or NADP^+^ as the electron acceptor, but its properties suggest a preferential use of NAD^+^ *in vivo*

The maximal specific activity of the recombinant enzyme depended on the electron acceptor used. With NADP^+^, a V_max_ of about 50 nkat (mg protein)^−1^ was found, corresponding to a catalytic constant of only 3 catalytic events s^−1^ *per* monomer (Table 2). With NAD^+^ instead of NADP^+^, a remarkably higher V_max_ value was obtained, corresponding to about 12 catalytic events s^−1^ for a single subunit. The use of either co-factor did not significantly change the affinity for the specific substrate, P5C, with *K*_M_ values of about 0.3 mM (Figure 7B). However, the affinity for NADP^+^ was strikingly lower than that for NAD^+^ (apparent *K*_M_ values of 0.6 mM and 5 mM for NAD^+^ or NADP^+^, respectively) (Figure 7A). Taking all these characteristics together, results clearly suggest that NAD^+^ most likely represents the preferred, if not the only co-factor used under *in vivo* conditions inside mitochondria.

#### Contrary to the bacterial protein, the native P5C dehydrogenase from rice is a homotetramer

**Figure 8:**
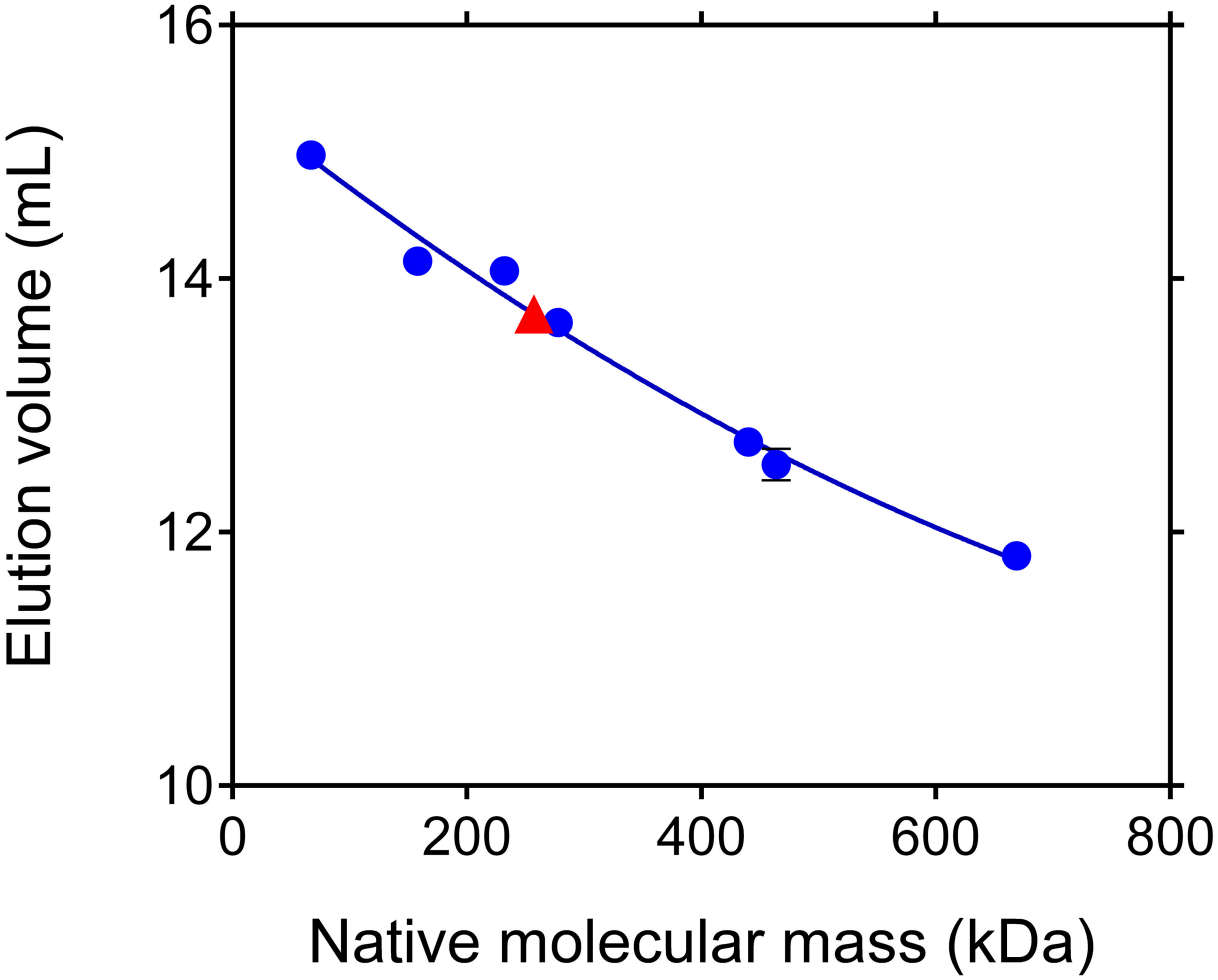
**Apparent molecular mass of rice P5C dehydrogenase under native conditions**. Aliquots (100 μL) of purified recombinant rice P5C dehydrogenase, adjusted to 0.5 mg mL^−1^, were subjected to gel permeation chromatography on a Superose 12 HR 10/30 (Pharmacia) column that had been equilibrated with 50 mM Tris-HCl buffer, pH 7.75, containing 250 mM NaCl. A molecular mass of 259 ± 14 kDa was estimated for P5C dehydrogenase, suggesting a homotetrameric composition of the enzyme. Molecular weight markers used (Pharmacia 17-0442-01 and 17-0441-01) are specified in Materials and Methods.

Recent data suggested that bacterial P5C dehydrogenases from *Thermus thermophilus* and *Deinococcus radiodurans* form trimer-of-dimers hexamers in solution, whereas the enzyme from *Bacillus* spp. forms dimers but does not assemble into higher-order oligomers (Luo et al., 2013). To verify whether the same may apply to rice P5C deydrogenase, its relative molecular mass was determined by gel filtration chromatography. Results were consistent with a native mass of 259 ± 14 kDa (Figure 8). The same pattern was obtained at different ionic strengths of the eluent in the range from 250 mM to 1 M NaCl, suggesting that the rice protein consists of 4 identical subunits, and does not disassemble into dimers.

#### P5C dehydrogenase is progressively inactivated by treatment at temperatures above 40°C, showing a biphasic profile of thermal inactivation

**Figure 9:**
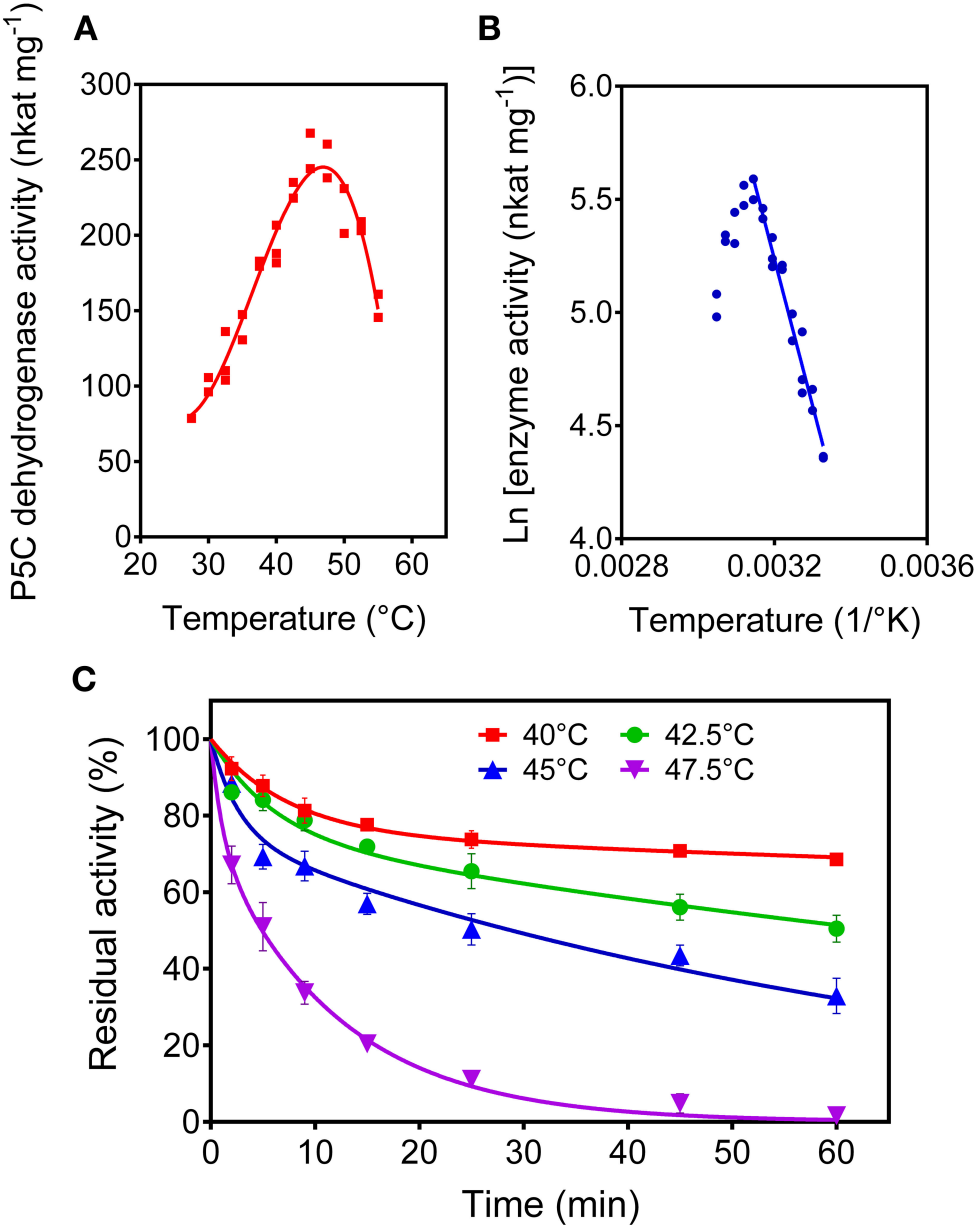
**Thermal stability of rice P5C dehydrogenase**. The activity rate of the purified enzyme was measured for up to 5 min under standard assay conditions at increasing temperatures **(A)**. Replotting data in the so-called Arrhenius plot **(B)** allowed the calculation of the activation energy (Table 2). Thermal stability of the enzyme was determined by incubating aliquots for increasing time at increasing temperature in the absence of substrates **(C)**. After the indicated times, the aliquots were immediately re-equilibrated on ice and the residual activity was then measured at 35°C, and expressed as percentage of activity in untreated controls. Three replicates were carried out for each treatment, and means ± SE over replicates are shown.

When the purified P5C dehydrogenase was assayed as a function of the temperature, maximal initial activity was obtained at a temperature as low as 46°C (Figure 9A). The corresponding activation energy, calculated by the Arrhenius plot (Figure 9B), was 54.6 ± 3.7 kJ mol^−1^. Moreover, the prolonged incubation of the rice protein at temperatures in the 40 to 50°C range in the absence of its substrates caused a dramatic and rapid loss of activity (Figure 9C). A treatment at 47.5°C for 5 min resulted in a 50%-inactivation of the enzyme. Inactivation curves did not follow the conventional one-phase exponential decay. The best fit of data was obtained assuming a two phase-decay with a very short (fast) and a moderate (slow) half-life. At 40°C, half-life (fast) and half-life (slow) were 5.2 ± 0.9 min and 507 ± 219 min, whereas at 45°C they were 1.8 ± 0.6 min and 49 ± 4 min, respectively.

#### P5C dehydrogenase activity is slightly sensitive to anions, whereas a variety of cations are inhibitory and appear to modulate the catalytic rate at physiological concentrations

**Figure 10:**
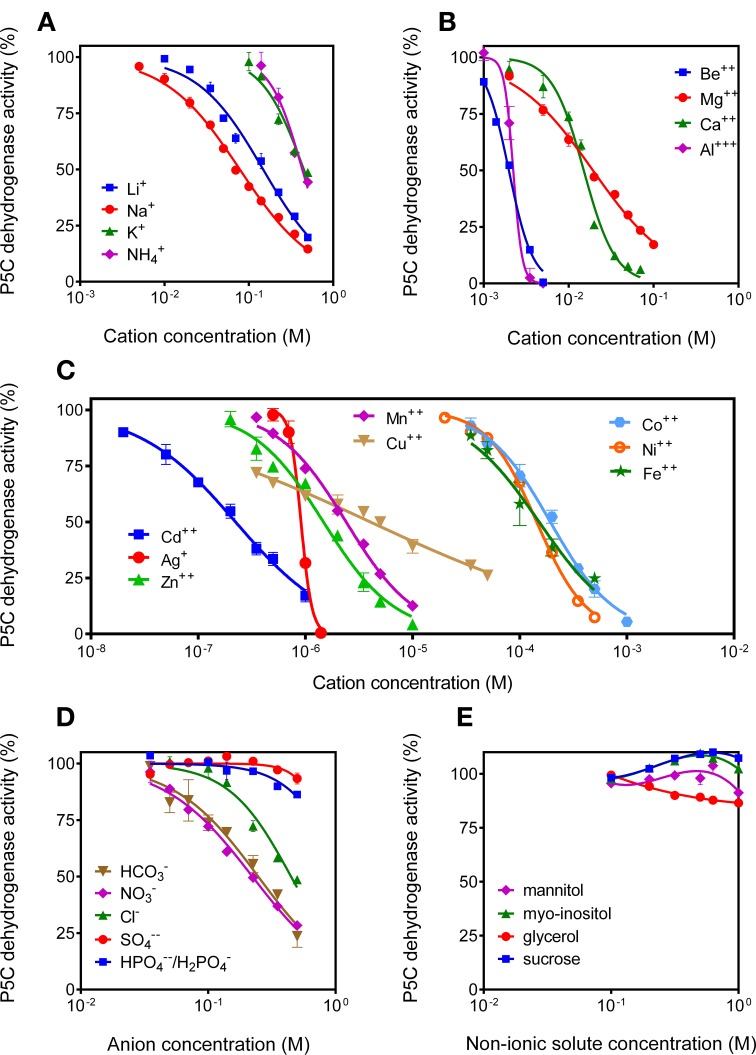
**Effect of anions and cations on the activity of P5C dehydrogenase**. The effects of the addition of increasing concentrations of monovalent cations **(A)**, divalent cations **(B)**, heavy metals **(C)**, various anions **(D)**, and non-ionic solutes **(E)** to the reaction mixture were assessed using NAD^+^ as the electron acceptor. Cations were added as chlorides, with the exception of Ca^2+^, Mg^2+^, Be^2+^, Cu^2+^, Zn^2+^, and Fe^2+^, which were added as sulfates. Anions were added as potassium salts. To minimize the carry-over of chloride anions, L-P5C concentration was reduced to 250 μM, and the P5C solution was neutralized just before use with 1 M NH_3_. Additionally, Hepes buffer was used at 12.5 mM to reduce the presence of potassium cations in the standard reaction mixture. In all cases, three replicates were carried out for each treatment. Data were expressed as percent of untreated controls, and mean values ± SE are presented.

Since P5C dehydrogenase from potato cultured cells had been found to be inhibited by NaCl concentrations above 100 mM (Forlani et al., 1997a), the possibility that high solute levels may reduce the activity of P5C dehydrogenase (and proline oxidation in turn) was considered. Because P5C neutralization and reaction mixture buffering may increase both anion and cation concentration in the assay environment, assay conditions were modified to limit the carry-over of ions. Under these conditions, the addition of non-ionic solutes up to 1 M to the reaction mixture was ineffective (Figure 10E). Anions exerted quite slight effects, and only bicarbonate and nitrate (but not chloride) were inhibitory at concentrations lower than 200 mM (Figure 10D). Interestingly, cations were much more potent inhibitors of P5C dehydrogenase. Li^+^ and Na^+^ progressively inhibited enzyme activity above 10 mM, whereas K^+^ and NH^+^^4^ were ineffective below 200 mM (Figure 10A). Divalent cations, also not considering the non-physiological Be^2+^ ions, were even more inhibitory, with a 32% reduction of the catalytic rate by Mg^2+^ at a concentration of 10 mM (Figure 10B). Very similar data were obtained when using NADP^+^ instead of NAD^+^ as the electron acceptor (not shown).

Interestingly, when the effect of a variety of other divalent cations was investigated, most were found to severely affect the activity of rice P5C dehydrogenase (Figure 10C). Some of them, as Cd^2+^ (IC_50_ of 2.2 × 10^−7^ M), may owe this ability to their oxidative properties, or are not of physiological significance. However, for others it does not seem the case, as they are normally present inside the plant cell and play a relevant role as micro-nutrients. Mainly for Zn^2+^ (IC_50_ of 1.5 × 10^−6^ M), Mn^2+^ (IC_50_ of 2.4 × 10^−6^ M), as well as Cu^2+^ (IC_50_ of 4.0 × 10^−6^ M), the possibility therefore exists that local concentrations in the mitochondria may be high enough to influence the catalytic rate of the enzyme.

#### Arginine inhibits P5C dehydrogenase activity with a mechanism of uncompetitive type with respect to P5C

**Figure 11:**
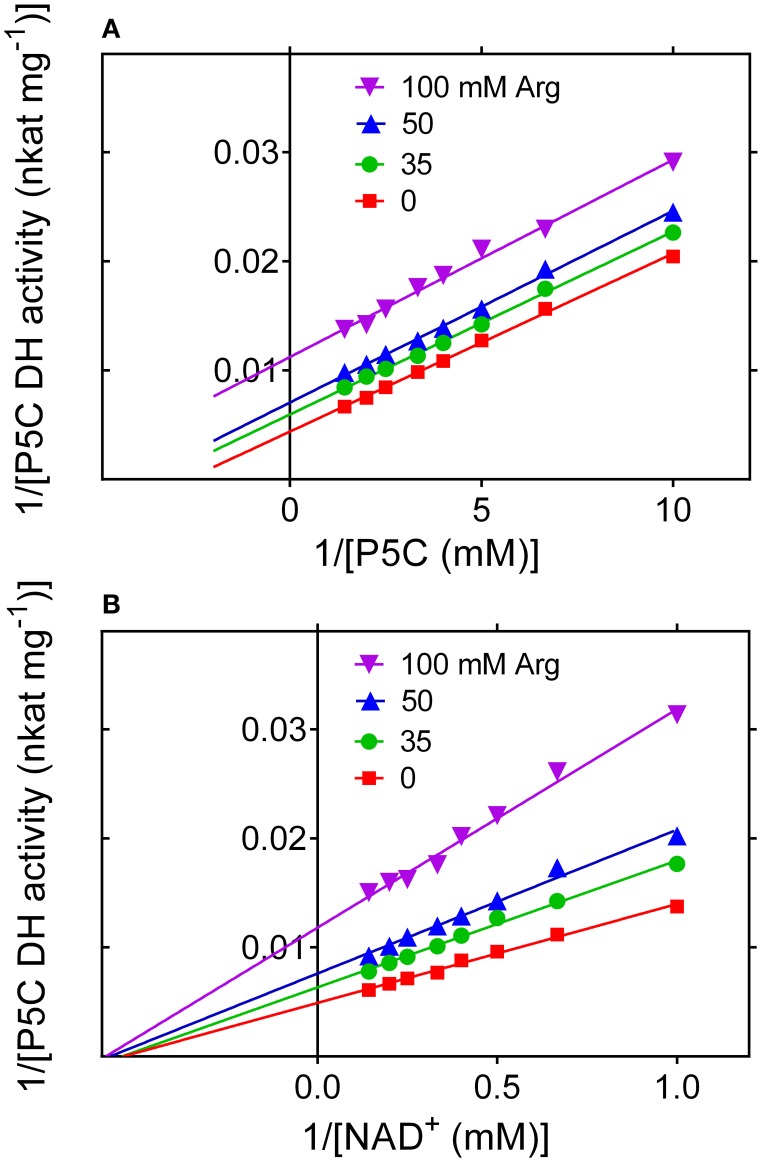
**Inhibition of P5C dehydrogenase by arginine**. The effect of increasing levels of arginine on the activity of the purified enzyme was determined using NAD^+^ as the electron acceptor at varying L-P5C **(A)** or NAD^+^ **(B)** concentration. Lines intersecting on the x axis account for a mechanism of non-competitive type with respect to NAD^+^ **(B)**, with a *K*_I_ value of 88.1 ± 2.9 mM. On the contrary, parallel lines for different arginine concentrations are consistent with an inhibition of uncompetitive type with respect to P5C **(A)**, with a *K*_I_ value of 62.3 ± 2.4 mM.

To evaluate whether the rice enzyme may be subjected to further post-translational regulative mechanisms, the activity of the purified protein was measured in the presence of increasing concentrations of proline, arginine, ornithine or glutamic acid. Since enzyme activity is strongly influenced by the pH in the 7 to 9 range (Figure 7C), special attention was paid to rule out the possibility that changes in the catalytic rate may depend on pH fluctuation induced by the addition of a given amino acid to the reaction mixture. Glutamate, proline and ornithine were substantially ineffective at concentrations ranging from 1 to 100 mM (data not presented). On the contrary, arginine was found to progressively inhibit the enzyme, with an IC_50_ value of 87 ± 11 mM. Citrulline was also inhibitory, but to a lower extent (not shown). Interestingly, a kinetic analysis showed that the inhibition by arginine is of non-competitive type with respect to NAD^+^, but of uncompetitive type with respect to P5C (Figure 11). The latter feature, quite uncommon, implies that in the presence of millimolar levels of arginine the K_*M*_ of P5C dehydrogenase for its specific substrate would be lowered, enhancing the binding of P5C even in the presence of low concentrations of the substrate.

## Discussion

### Rice P5C dehydrogenase is constitutively expressed and high intracellular levels of proline increase only slightly its mRNA abundance, whereas its transcript level seems not subject to environmental control

Proline oxidation to glutamate takes place in the mitochondrion by means of a short pathway in which a membrane-bound proline dehydrogenase converts proline into P5C, which in turn is further oxidized by a soluble P5C dehydrogenase (Forlani et al., 1997a). P5C can be alternatively produced from ornithine by OAT, thus P5C dehydrogenase is involved also in arginine catabolism (Winter et al., 2015). Export of P5C from mitochondria in a proline-P5C cycle has also been postulated (Miller et al., 2009). In this case, the balance between export and P5C dehydrogenase activity would be decisive about the further fate of organic nitrogen and carbon derived from proline and arginine catabolism. Although P5C dehydrogenase is usually considered as non-rate-limiting in proline and arginine catabolism, its fine control may be important to balance the two pathways and to determine the utilization of P5C. Very little is known about the occurrence of post-translational regulatory mechanisms for plant P5C dehydrogenases, whereas some studies described fluctuations of transcript levels in response to either exogenous proline, with maximal expression 48 h after the treatment (Deuschle et al., 2001), or to ABA treatment and hyperosmotic stress conditions induced by PEG (but not by salt; Gao and Han, 2009). However, in the latter case a 2-fold induction of the transcript level was in apparent contrast with the need to accumulate proline to withstand stress conditions. In all cases, intracellular free proline content had not been determined and related to expression levels.

The results obtained in the present study showed the presence of transcripts of *OsP5CDH*, the apparently only gene coding for a P5C dehydrogenase in rice, in cell cultures under all the conditions tested. *OsP5CDH* transcript levels were generally low in comparison to those of the anabolic *P5C reductase* and *P5C synthetase 2*, and no significant variations were found in response to either salt or drought stress, ABA treatment or exogenous administration of proline (or arginine), despite significant fluctuations of endogenous free proline (or arginine) levels during some of these treatments. Merely 24 h after the exposure to high PEG concentrations, when proline content had already doubled over control levels, a two-fold increase of the *OsP5CDH* transcript level was evident. Interestingly, also the relative normalized expression of the genes coding for the enzymes involved in proline biosynthesis did not show remarkable variations in response to PEG treatments, suggesting that some fluctuation of free proline content may arise without the need of transcriptional control. Also the detailed characterization of P5C reductase activity patterns recently suggested post-translational control of fluxes in proline metabolism (Giberti et al., 2014; Forlani et al., 2015). Nevertheless, normalized expression levels of *Proline dehydrogenase* were found almost undetectable in controls, but showed a strong increase in response to exogenous proline. On the contrary, *OAT* transcript levels were similarly low under control conditions but were not enhanced by treatment with exogenous arginine. On the whole, it seems therefore that P5C dehydrogenase is expressed constitutively at levels high enough to allow the utilization of even highest concentrations of P5C generated by proline and arginine degradation. However, in this overall picture it is unclear which may be the role of P5C dehydrogenase in actively proliferating cells under normo-osmotic conditions, when proline dehydrogenase is virtually unexpressed and arginine accounts for less than 0.5% of total free amino acids. In several cereals, the expression of the genes coding for P5C dehydrogenase was almost undetectable in leaves, but showed hundredfold induction following the infection with a compatible rust strain (Ayliffe et al., 2002). On the contrary, a pathogen-induced early induction of *Proline dehydrogenase* transcript levels in *A. thaliana* was accompanied by an increase in *P5C reductase*, but not in *AtP5CDH* transcripts (Cecchini et al., 2011). These apparently inconsistent data may reflect differences among plant species, or depend on the interaction with specific pathogens. In any case, the results obtained by RT-PCR for *OsP5CDH* expression levels in rice cell cultures are in agreement with previous Northern blot data on *A. thaliana* seedlings (Deuschle et al., 2001) and Western blot analysis of potato tissues (Forlani et al., 2000). In the latter study, protein levels were also suggestive of a developmental, but not of an environmental control of P5C dehydrogenase expression in response to abiotic stress.

Taken together, all these elements could imply that an inverse transcriptional control of proline dehydrogenase and P5C synthetase is sufficient to determine the suitable intracellular concentration of proline. However, it has been shown that in *A. thaliana* the need to limit proline oxidation under salt and drought stress conditions requires an at least partial silencing of *AtP5CDH* expression *via* naturally occurring siRNAs derived from induction of the overlapping *SRO5* gene (Borsani et al., 2005). Nevertheless, this does not seem to be a universal system, since no genes partially overlapping with *OsP5CDH* are annotated in rice, and a BLAST search with the *A. thaliana SRO5* (either nucleotide or amino acid) sequence did not show the presence of any ortholog (*E*-value > 0.2 in BLAST-N) in the rice genome. Even in the absence of control mechanisms at both the transcriptional and the translational level, a modulation of the rate of P5C oxidation may derive from post-translational regulation of enzyme activity.

### Rice P5C dehydrogenase is a quite labile, homotetrameric enzyme with a low apparent catalytic efficiency and a clear-cut preference for NAD^+^ as the electron acceptor

The assessment of post-translational regulation of P5C dehydrogenase activity has been hampered to date by a substantial lack of information about the biochemical properties of the enzyme from higher plants. In early studies, enzyme activity was detected in crude mitochondrial extracts, and a preliminary characterization carried out with partially purified preparations pointed out a preference for NAD^+^ over NADP^+^ as the electron acceptor and *K*_M_ values in the micromolar to millimolar range for both substrates (Stewart and Lai, 1974; Boggess et al., 1975). So far, the plant enzyme has been purified and kinetically characterized only from potato (Forlani et al., 1997a). The results obtained with the recombinant rice P5C dehydrogenase provide some confirmation, and add important new information on activity modulation according to the physiological status of the cell. Similarly to the potato enzyme, rice P5C dehydrogenase showed affinity constants between 10^−4^ M and 10^−3^ M for both P5C and NAD^+^, i.e., values very close to the intracellular concentrations of P5C (10 nmol to 20 nmol [g fresh weigh]^−1^; Forlani et al., 2013) and the preferred electron acceptor NAD^+^ (16 nmol [g fresh weigh]^−1^; Hayashi et al., 2005). This implies that inside the cell the catalytic rate may be much lower than the V_*max*_ measured *in vitro*. The catalytic constant of 12 s^−1^ suggests that P5C dehydrogenase is an enzyme with a rather poor efficiency. However, *K*_M_ values in the range of the actual substrate concentrations facilitate the attainment of homeostatic levels, since any increase of substrate concentrations would immediately cause a corresponding increase of enzyme activity. Moreover, the evaluation of the temperature-activity relationship pointed out a poor thermal stability of P5C dehydrogenase, which in the absence of its substrates was rapidly and irreversibly inactivated at temperatures exceeding 40°C, and lost more than 25% activity in 1 month even when stored at 4°C. As a term of comparison, *A. thaliana* P5C reductase showed a catalytic constant of about 700 s^−1^ (Giberti et al., 2014), and retained more than 50% activity after 3 weeks at room temperature (Funck et al., 2012). In the presence of substrates, the effect was slightly milder, but temperatures exceeding 46°C rapidly led to activity loss of rice P5C dehydrogenase. A protective effect of substrates against thermal inactivation has been reported for other enzymes in amino acid metabolism (e.g., Forlani, 1997). The low catalytic efficiency and fast turn-over rate of P5C dehydrogenase may explain at least in part the need to maintain relatively high constitutive transcript levels.

On the other hand, size exclusion chromatography experiments clearly supported a homotetrameric composition of recombinant rice P5C dehydrogenase. This confirms previous data on the potato enzyme, and indicates a different oligomeric composition of plant P5C dehydrogenases compared to the corresponding bacterial enzymes. The latter have been recently shown to form dimers that do not assemble into higher-order oligomers, or form trimer-of-dimers hexamers in solution (Luo et al., 2013). No evidence supporting the existence of free dimers has been obtained for the rice protein, since elution patterns upon gel filtration were unaffected by the addition of up to 1 M NaCl to the eluent. The enzyme from plants seems therefore to possess a structure significantly different from either the bifunctional proline oxidase present in enterobacteria and bearing both proline- and P5C-dehydrogenase activities, or the monofunctional P5C dehydrogenase from archea and Gram positive eubacteria.

### Several parameters, including pH and cation concentrations, regulate the activity of rice P5C dehydrogenase

Interestingly, both the presence of salts and changes of pH in the physiological range were found to greatly influence the catalytic rate of purified rice P5C dehydrogenase. Maximal activity was shown between pH 6.6 and pH 7, and a progressive loss of efficiency was evident along with the alkalinization of the reaction mixture, with about 50% activity at pH 8.0 and almost no activity at pH 9.0. This activity pattern is most probably a superimposition of the intrinsic enzyme properties and the equilibrium in solution between P5C and GSA, which seems to be the actual substrate of P5C dehydrogenase (Arentson et al., 2012). The GSA/P5C ratio shows a very sharp transition around pH 6.2, with GSA being the prevalent form in acidic conditions (Bearne and Wolfenden, 1995). The peak of P5C dehydrogenase activity between pH 6.8 and pH 7 may thus reflect the optimum between GSA concentration and protonation dependent inactivation of the enzyme, while the shoulder at pH 8 may reflect the optimal pH for catalysis. Recently, transient partial depolarization of the inner mitochondrial membrane has been reported to occur in *A. thaliana* in response to high temperature, H_2_O_2_, or cadmium treatments. Membrane potential pulses were coincident with a pronounced transient alkalinization (up to >1 pH unit) of the matrix (Schwarzländer et al., 2012). The authors proposed a role for pulsing as a transient uncoupling mechanism to counteract mitochondrial dysfunction and reactive oxygen species production. However, this might also reduce P5C (and proline) oxidation under oxidative stress conditions, when proline may serve as a radical scavenger.

Moreover, several cations were found to inhibit rice P5C dehydrogenase, whereas anions were effective only at highest, mostly unphysiological levels. Previous data accounting for a strong inhibition by chlorides (e.g., Boggess et al., 1975) could most likely be a consequence of the carry-over of Cl^−^ ions from P5C solution to the standard assay mixture. Based on our results, a salt stress-driven increase of the intracellular concentration of Na^+^ cations from 10 to 100 mM would result in a progressive reduction of P5C oxidation, possibly contributing to proline accumulation. The same seems true for Mg^2+^: since Mg^2+^concentration has been found remarkably constant and low in the cytosol but tenfold higher in the mitochondrial matrix, where its fluctuation might regulate cell respiration (Gout et al., 2014), also this feature might be of physiological significance. Even more interestingly, rice P5C dehydrogenase showed severe inhibition by a wide array of divalent cations. In some cases, cation inhibition is unlikely to occur *in vivo*, since ions like Cd^2+^ are not usually present inside living cells. In other cases, on the contrary, these effects could allow environmental factors to modulate P5C oxidation. For instance, iron, copper, and manganese have been detected at micromolar levels in higher plant mitochondria, and some evidence has been described supporting the possibility that their fluctuations may modulate the activity of several mitochondrial enzymes (Tan et al., 2010). Once again, such variations were found following *in vivo* and *in vitro* oxidative stresses, conditions that could prompt proline accumulation and therefore require modulation of the degradation pathway.

### High concentrations of free arginine may increase the affinity of P5C dehydrogenase for its specific substrate, possibly influencing the balance between P5C export into the cytosol and its oxidation to glutamate

The presence of amino acids of the glutamate family at physiological concentrations did not significantly influence P5C dehydrogenase activity, except for arginine. In the 10 to 100 mM range, arginine was found to progressively inhibit the activity of the purified enzyme. Inhibition of P5C dehydrogenase by high concentrations of arginine seems inconsistent with a role of the enzyme in arginine utilization. High intracellular levels of arginine have been described in germinating seeds of various species, where arginine degradation supplies organic and NH^+^^4^ nitrogen for anabolic processes (Winter et al., 2015). To allow the synthesis of nitrogen-containing compounds needed for growth, arginine is converted into urea and glutamate, the latter serving as the main donor of amino mojeties. However, the mechanism of enzyme inhibition shed some light on this apparent discrepancy. Being of uncompetitive type with respect to P5C, the inhibition brought about by arginine would be most effective in the presence of saturating levels of P5C, which on the contrary is maintained at very low levels inside the cell to avoid cytotoxic effects (Deuschle et al., 2004). In the presence of limiting P5C concentrations, the interaction with arginine results in a higher affinity for P5C. For instance, in the presence of 35 or 50 mM arginine, the K_M(app)_ for l-P5C_(NAD^+^)_ is reduced from 358 μM (Table 2) to 270 and 235 μM, respectively. Since P5C levels of no more than 10–20 nmol (g fresh weigh)^−1^ have been found in plant cell cultures (Forlani et al., 2013), this would result in a stimulation of P5C oxidation to glutamate over its transfer into the cytosol to fuel the P5C/proline cycle. Further data will be required to obtain conclusive evidence supporting the occurrence of this mechanism *in vivo*. However, because of the structural difference between arginine and P5C, and the lack of a similar effect in the case of ornithine, it seems unlikely that the presence of allosteric effects of arginine (and citrulline, although to a lower extent) on P5C dehydrogenase activity may be purely casual.

The occurrence of two enzyme forms, as reported for *N. plumbaginifolia* P5C dehydrogenase (Forlani et al., 1997b), would provide an efficient way to regulate separately arginine and proline catabolism. The discrimination between proline- and arginine-derived P5C could in this case be achieved *in vivo* by protein-protein interactions, with P5C dehydrogenases being associated with either proline dehydrogenase or OAT (Elthon and Stewart, 1982). However, in several if not in most diploid plant species only a single gene seems to exist, and so far no direct evidence for different splicing variants or protein modifications was reported (e.g., Deuschle et al., 2001). As a consequence, multiple regulative mechanisms are required to modulate the activity of a single P5C dehydrogenase in response to either the activation of proline or arginine catabolism, or the need to limit proline oxidation to cope with abiotic stress conditions.

The results described in this work indicate that P5C dehydrogenase activity might be highly sensitive to metabolite or ion concentration changes in the micro-environment of the enzyme, and may well have a regulatory impact on the rates or routes of P5C metabolism. For instance, during seed germination, the hydrolysis of arginine-rich seed proteins increases free arginine in the cell to millimolar levels: this in turn would increase the affinity of P5C dehydrogenase to P5C, favoring its oxidation to glutamate. On the other hand, under either oxidative or salt stress conditions, increased mitochondrial levels of sodium ions and heavy metals might limit P5C oxidation, possibly facilitating the P5C/Pro cycle (Miller et al., 2009). Conversely, their lowering to homeostatic concentrations would favor proline oxidation under the subsequent recovery from stress conditions. In the next future we plan to verify our results at the plant level, on both salt stress sensitive and tolerant rice genotypes, and see whether different expression levels and/or properties of P5C dehydrogenase may correlate with a differential susceptibility to excess salt.

### Conflict of interest statement

The authors declare that the research was conducted in the absence of any commercial or financial relationships that could be construed as a potential conflict of interest.
