# Supplementary material for: Functional characterization and expression analysis of rice δ1-pyrroline-5-carboxylate dehydrogenase provide new insight into the regulation of proline and arginine catabolism
Source: Front Plant Sci. 2015 Aug 5;6:591. doi: 10.3389/fpls.2015.00591 (PMC4525382; doi:10.3389/fpls.2015.00591)
Supplement: Supplementary file 1 [file FigureS1.PDF]

A *OsP5CDH* CDS and expression clones

```
J033091O16-ORF     ------------------------------------------------------------ 0
pET151-OsP5CDH     ATGCATCATCACCATCACCATGGTAAGCCTATCCCTAACCCTCTCCTCGGTCTCGATTCT 60
pET151-ntOsP5CDH   ATGCATCATCACCATCACCATGGTAAGCCTATCCCTAACCCTCTCCTCGGTCTCGATTCT 60

J033091O16-ORF     ---------------------------------------ATGAGCCTCATCCTTTCCCGG 21
pET151-OsP5CDH     ACGGAAAACCTGTATTTTCAGGGAATTGATCCCTTCACC--------------------- 99
pET151-ntOsP5CDH   ACGGAAAACCTGTATTTTCAGGGAATTGATCCCTTCACCATGAGCCTCATCCTTTCCCGG 120

J033091O16-ORF     CGGCGCCTCGCCGCCGCCGTCCGGCGGTCAGGCCCCGCCGCGCTCGCCTCCAGGTGGATG 81
pET151-OsP5CDH     ---------------------------TCAGGTCCAGCCGCACTCGCCTCCAGGTGGATG 132
pET151-ntOsP5CDH   CGGCGCCTC---------------------------GCCGCGCTCGCCTCCAGGTGGATG 153
                                                    ***** ******************

J033091O16-ORF     CACACGCCGCCATTCGCGACGGTGTCTCCTCAGGAAATTTCAGGTTCGAGCCCGGCGGAA 141
pET151-OsP5CDH     CACACGCCGCCATTCGCGACGGTGTCTCCTCAGGAAATTTCAGGTTCGAGCCCGGCGGAA 192
pET151-ntOsP5CDH   CACACGCCGCCATTCGCGACGGTGTCTCCTCAGGAAATTTCAGGTTCGAGCCCGGCGGAA 213
                   ************************************************************

J033091O16-ORF     GTGCAGAATTTTGTGCAGGGCAGTTGGACAACATCTGGTAACTGGAATTGGCTAGTTGAT 201
pET151-OsP5CDH     GTGCAGAATTTTGTGCAGGGCAGTTGGACAACATCTGGTAACTGGAATTGGCTAGTTGAT 252
pET151-ntOsP5CDH   GTGCAGAATTTTGTGCAGGGCAGTTGGACAACATCTGGTAACTGGAATTGGCTAGTTGAT 273
                   ************************************************************

J033091O16-ORF     CCTTTAAATGGTGAAAAATTTATCAAAGTTGCTGAGGTTCAGGAAGCAGAAATAAAGCCA 261
pET151-OsP5CDH     CCTTTAAATGGTGAAAAATTTATCAAAGTTGCTGAGGTTCAGGAAGCAGAAATAAAGCCA 312
pET151-ntOsP5CDH   CCTTTAAATGGTGAAAAATTTATCAAAGTTGCTGAGGTTCAGGAAGCAGAAATAAAGCCA 333
                   ************************************************************

J033091O16-ORF     TTTGTAGAGAGTTTATCTAATTGCCCAAAGCATGGACTTCACAACCCACTTAAAGCTCCA 321
pET151-OsP5CDH     TTTGTAGAGAGTTTATCTAATTGCCCAAAGCATGGACTTCACAACCCACTTAAAGCTCCA 372
pET151-ntOsP5CDH   TTTGTAGAGAGTTTATCTAATTGCCCAAAGCATGGACTTCACAACCCACTTAAAGCTCCA 393
                   ************************************************************

J033091O16-ORF     GAGAGGTATCTCATGTATGGAGATATATCTGCCAAAGCTGCAAACATGCTTGGTCAACCT 381
pET151-OsP5CDH     GAGAGGTATCTCATGTATGGAGATATATCTGCCAAAGCTGCAAACATGCTTGGTCAACCT 432
pET151-ntOsP5CDH   GAGAGGTATCTCATGTATGGAGATATATCTGCCAAAGCTGCAAACATGCTTGGTCAACCT 453
                   ************************************************************

J033091O16-ORF     GTGGTTTCAGATTTCTTTGCTAAACTTATCCAGAGGGTATCTCCAAAGAGCTATCAGCAA 441
pET151-OsP5CDH     GTGGTTTCAGATTTCTTTGCTAAACTTATCCAGAGGGTATCTCCAAAGAGCTATCAGCAA 492
pET151-ntOsP5CDH   GTGGTTTCAGATTTCTTTGCTAAACTTATCCAGAGGGTATCTCCAAAGAGCTATCAGCAA 513
                   ************************************************************

J033091O16-ORF     GCTCTTGCAGAAGTTCAAGTCTCTCAAAAAATTCTTGGAGAACTTTTGTGGAGATCAGGTA 501
pET151-OsP5CDH     GCTCTTGCAGAAGTTCAAGTCTCTCAAAAAATTCTTGGAGAACTTTTGTGGAGATCAGGTA 552
pET151-ntOsP5CDH   GCTCTTGCAGAAGTTCAAGTCTCTCAAAAAATTCTTGGAGAACTTTTGTGGAGATCAGGTA 573
                   ************************************************************

J033091O16-ORF     CGCTTTCTGGCTCGATCATTTGCTGTACCTGGCAACCATCTTGGACAAAGCAGTAATGGT 561
pET151-OsP5CDH     CGCTTTCTGGCTCGATCATTTGCTGTACCTGGCAACCATCTTGGACAAAGCAGTAATGGT 612
pET151-ntOsP5CDH   CGCTTTCTGGCTCGATCATTTGCTGTACCTGGCAACCATCTTGGACAAAGCAGTAATGGT 633
                   ************************************************************

J033091O16-ORF     TACCGTTGGCCATATGGTCCGGTTGCAATTATCACACCGTTCAATTTCCCATTGGAGATT 621
pET151-OsP5CDH     TACCGTTGGCCATATGGTCCGGTTGCAATTATCACACCGTTCAATTTCCCATTGGAGATT 672
pET151-ntOsP5CDH   TACCGTTGGCCATATGGTCCGGTTGCAATTATCACACCGTTCAATTTCCCATTGGAGATT 693
                   ************************************************************

J033091O16-ORF     CCATTACTGCAACTAATGGGAGCACTGTATATGGGAAATAAACCTGTCCTCAAAGTTGAC 681
pET151-OsP5CDH     CCATTACTGCAACTAATGGGAGCACTGTATATGGGAAATAAACCTGTCCTCAAAGTTGAC 732
pET151-ntOsP5CDH   CCATTACTGCAACTAATGGGAGCACTGTATATGGGAAATAAACCTGTCCTCAAAGTTGAC 753
                   ************************************************************

J033091O16-ORF     AGCAAAGTTAGCATTGTGATGGACCAGATGCTTAGGTTGCTTCACGCATGTGGGATGCCA 741
pET151-OsP5CDH     AGCAAAGTTAGCATTGTGATGGACCAGATGCTTAGGTTGCTTCACGCATGTGGGATGCCA 792
pET151-ntOsP5CDH   AGCAAAGTTAGCATTGTGATGGACCAGATGCTTAGGTTGCTTCACGCATGTGGGATGCCA 813
                   ************************************************************

J033091O16-ORF     GCAGAGGATGTGGACTTCATAAATTCTGATGGTATCACGATGAACAAGCTGCTGTTAGAG 801
pET151-OsP5CDH     GCAGAGGATGTGGACTTCATAAATTCTGATGGTATCACGATGAACAAGCTGCTGTTAGAG 852
pET151-ntOsP5CDH   GCAGAGGATGTGGACTTCATAAATTCTGATGGTATCACGATGAACAAGCTGCTGTTAGAG 873
                   ************************************************************
```

```
J033091016-ORF    GCAAATCCGAAAATGACCCTCTTCACTGGGAGCTCACGGATAGCAGAGAAATTGGCTGCT 861
pET151-OsP5CDH    GCAAATCCGAAAATGACCCTCTTCACTGGGAGCTCACGGATAGCAGAGAAATTGGCTGCT 912
pET151-ntOsP5CDH  GCAAATCCGAAAATGACCCTCTTCACTGGGAGCTCACGGATAGCAGAGAAATTGGCTGCT 933
                  ************************************************************

J033091016-ORF    GATTTGAAAGGCAAAATCAAATTGGAAGATGCTGGTTTTGACTGGAAAATTCTTGGTCCA 921
pET151-OsP5CDH    GATTTGAAAGGCAAAATCAAATTGGAAGATGCTGGTTTTGACTGGAAAATTCTTGGTCCA 972
pET151-ntOsP5CDH  GATTTGAAAGGCAAAATCAAATTGGAAGATGCTGGTTTTGACTGGAAAATTCTTGGTCCA 993
                  ************************************************************

J033091016-ORF    GATGTTCAAGAGGTTGATTACATTGCATGGGTTTGCGACCAGGATGCTTATGCTTGCAGT 981
pET151-OsP5CDH    GATGTTCAAGAGGTTGATTACATTGCATGGGTTTGCGACCAGGATGCTTATGCTTGCAGT 1032
pET151-ntOsP5CDH  GATGTTCAAGAGGTTGATTACATTGCATGGGTTTGCGACCAGGATGCTTATGCTTGCAGT 1053
                  ************************************************************

J033091016-ORF    GGTCAGAAGTGCTCTGCTCAGTCTATTCTATTCATGCACAAGAATTGGTCGTCTAGTGGG 1041
pET151-OsP5CDH    GGTCAGAAGTGCTCTGCTCAGTCTATTCTATTCATGCACAAGAATTGGTCGTCTAGTGGG 1092
pET151-ntOsP5CDH  GGTCAGAAGTGCTCTGCTCAGTCTATTCTATTCATGCACAAGAATTGGTCGTCTAGTGGG 1113
                  ************************************************************

J033091016-ORF    CTTCTTGATAAAATGAAAAGTCTTTCTGAAAGAAGGAAGCTTGAAGACTTGACCATTGGC 1101
pET151-OsP5CDH    CTTCTTGATAAAATGAAAAGTCTTTCTGAAAGAAGGAAGCTTGAAGACTTGACCATTGGC 1152
pET151-ntOsP5CDH  CTTCTTGATAAAATGAAAAGTCTTTCTGAAAGAAGGAAGCTTGAAGACTTGACCATTGGC 1173
                  ************************************************************

J033091016-ORF    CCAGTCCTTACTGTTACAACATCAAGCATGATAGAGCACATGAAAAACCTTCTCAAAATA 1161
pET151-OsP5CDH    CCAGTCCTTACTGTTACAACATCAAGCATGATAGAGCACATGAAAAACCTTCTCAAAATA 1212
pET151-ntOsP5CDH  CCAGTCCTTACTGTTACAACATCAAGCATGATAGAGCACATGAAAAACCTTCTCAAAATA 1233
                  ************************************************************

J033091016-ORF    CCAGGATCAAAGGTCCTGTTTGGTGGTGAACCTCTGGAGAACCATTCTATCCCAGAAATA 1221
pET151-OsP5CDH    CCAGGATCAAAGGTCCTGTTTGGTGGTGAACCTCTGGAGAACCATTCTATCCCAGAAATA 1272
pET151-ntOsP5CDH  CCAGGATCAAAGGTCCTGTTTGGTGGTGAACCTCTGGAGAACCATTCTATCCCAGAAATA 1293
                  ************************************************************

J033091016-ORF    TATGGTGCCTTCAAACCAACTGCTGTATTTGTTCCTCTATCGGAAATCCTTAAAAGTGGC 1281
pET151-OsP5CDH    TATGGTGCCTTCAAACCAACTGCTGTATTTGTTCCTCTATCGGAAATCCTTAAAAGTGGC 1332
pET151-ntOsP5CDH  TATGGTGCCTTCAAACCAACTGCTGTATTTGTTCCTCTATCGGAAATCCTTAAAAGTGGC 1353
                  ************************************************************

J033091016-ORF    AATTTTGAGCTTGTGACAAGGGAGATCTTTGGTCCTTTCCAGGTGGTTACAGAGTATTCT 1341
pET151-OsP5CDH    AATTTTGAGCTTGTGACAAGGGAGATCTTTGGTCCTTTCCAGGTGGTTACAGAGTATTCT 1392
pET151-ntOsP5CDH  AATTTTGAGCTTGTGACAAGGGAGATCTTTGGTCCTTTCCAGGTGGTTACAGAGTATTCT 1413
                  ************************************************************

J033091016-ORF    GATGATGAGCTTGAATTAGTATTAGAAGCCTGTGAAAGGATGAACGCTCATCTGACGGCT 1401
pET151-OsP5CDH    GATGATGAGCTTGAATTAGTATTAGAAGCCTGTGAAAGGATGAACGCTCATCTGACGGCT 1452
pET151-ntOsP5CDH  GATGATGAGCTTGAATTAGTATTAGAAGCCTGTGAAAGGATGAACGCTCATCTGACGGCT 1473
                  ************************************************************

J033091016-ORF    GCAGTTGTTTCAAACGACCCTTTATTCCTGCAGGAAGTACTTGGGCGATCAGTTAACGGG 1461
pET151-OsP5CDH    GCAGTTGTTTCAAACGACCCTTTATTCCTGCAGGAAGTACTTGGGCGATCAGTTAACGGG 1512
pET151-ntOsP5CDH  GCAGTTGTTTCAAACGACCCTTTATTCCTGCAGGAAGTACTTGGGCGATCAGTTAACGGG 1533
                  ************************************************************

J033091016-ORF    ACAACATATGCCGGCATCCGAGCAAGGACAACTGGTGCTCCACAGAACCACTGGTTTGGG 1521
pET151-OsP5CDH    ACAACATATGCCGGCATCCGAGCAAGGACAACTGGTGCTCCACAGAACCACTGGTTTGGG 1572
pET151-ntOsP5CDH  ACAACATATGCCGGCATCCGAGCAAGGACAACTGGTGCTCCACAGAACCACTGGTTTGGG 1593
                  ************************************************************

J033091016-ORF    CCTGCTGGTGATCCAAGAGGTGCAGGGATCGGAACTCCAGAAGCCATTAAACTCGTTTGG 1581
pET151-OsP5CDH    CCTGCTGGTGATCCAAGAGGTGCAGGGATCGGAACTCCAGAAGCCATTAAACTCGTTTGG 1632
pET151-ntOsP5CDH  CCTGCTGGTGATCCAAGAGGTGCAGGGATCGGAACTCCAGAAGCCATTAAACTCGTTTGG 1653
                  ************************************************************

J033091016-ORF    TCTTGCCACAGGGAGATCATATATGACATTGGCCCCTTGCCTAAGAACCGGGCACTACCT 1641
pET151-OsP5CDH    TCTTGCCACAGGGAGATCATATATGACATTGGCCCCTTGCCTAAGAACCGGGCACTACCT 1692
pET151-ntOsP5CDH  TCTTGCCACAGGGAGATCATATATGACATTGGCCCCTTGCCTAAGAACCGGGCACTACCT 1713
                  ************************************************************

J033091016-ORF    TCCGCTACATAAATACTATGTCAAGGCTAGATTAGCTTGTAGCATGTTATTC   1693
pET151-OsP5CDH    TCCGCTACATAAATACTATGTCAAGGCTAGATTAGCTTGTAGCAAGGGCGAG   1744
pET151-ntOsP5CDH  TCCGCTACATAAATACTATGTCAAGGCTAGATTAGCTTGTAGCAAGGGCGAG   1765
                  ******************************************** *
```

ATG start codon   stop codon   Primers (Fwd-Rev)

B Recombinant and native *Os*P5CDH and confirmed peptides

polyhistidine tag   V5 epitope   TEV cleavage site   transit peptide

```
Native OsP5CDH -----------------MSLILSRRRLAAAVRRSGPAALASRWMHTPPFATVSPQEISGS  43
Recomb OsP5CDH MHHHHHHGKPIPNPLLGLDSTENLYFQGIDPFTSGPAALASRWMHTPPFATVSPQEISGS  60
Pepsin         -------------PLLGLDSTENLYFQGIDPFTSGPAALASRWMHTPPFATVSPQEISGS
Trypsin        ------------------------------------------------------------

Recomb OsP5CDH SPAEVQNFVQGSWTTSGNWNWLVDPLNGEKFIKVAEVQEAEIKPFVESLSNCPKHGLHNP 120
Pepsin         SPAEVQNFVQGSWTTSGNWNWLVDPLNGEKFIKVAEVQEAEIKPFVESLSNCPKHGLHNP
Trypsin        ---------------------------------VAEVQEAEIKPFVESLSNCPKHGLHNP

Recomb OsP5CDH LKAPERYLMYGDISAKAANMLGQPVVSDFFAKLIQRVSPKSYQQALAEVQVSQKFLENFC 180
Pepsin         LKAPERYLMYGDISAKAANMLGQPVVSDF-AKLIQRVSPKSYQQALAEVQVSQKFLENFC
Trypsin        LK----YLMYGDISAKAANMLGQPVVSDFFAKLIQR----SYQQALAEVQVSQKFLENFC

Recomb OsP5CDH GDQVRFLARSFAVPGNHLGQSSNGYRWPYGPVAIITPFNFPLEIPLLQLMGALYMGNKPV 240
Pepsin         GDQVRFLARSFAVPGNHLGQSSNGYRWPYGPVAIITPFNFPLEIPLLQLMGALYMGNKPV
Trypsin        GDQVR----SFAVPGNHLGQSSNGYRWPYGPVAIITPFNFPLEIPLLQLMGALYMGNKPV

Recomb OsP5CDH LKVDSKVSIVMDQMLRLLHACGMPAEDVDFINSDGITMNKLLLEANPKMTLFTGSSRIAE 300
Pepsin         LKVDSKVSIVMDQMLRLLHACGMPAEDVDFINSDGITMNKLLLEANPKMTLFTGSSRIAE
Trypsin        LKVDSKVSIVMDQMLRLLHACGMPAEDVDFINSDGITMNKLLLEANPKMTLFTGSSR---

Recomb OsP5CDH KLAADLKGKIKLEDAGFDWKILGPDVQEVDYIAWVCDQDAYACSGQKCSAQSILFMHKNW 360
Pepsin         KLAADLKGKIKLEDAGFDWKILGPDVQEVDYIAWVCDQDA--------------FMHKNW
Trypsin        ---------IKLEDAGFDWKILGPDVQEVDYIAWVCDQDAYACSGQKCSAQSILFMHKNW

Recomb OsP5CDH SSSGLLDKMKSLSERRKLEDLTIGPVLTVTTSSMIEHMKNLLKIPGSKVLFGGEPLENHS 420
Pepsin         SSSGLLDKMKSLSERRKLEDLTIGPVLTVTTSSMIEHMKNLLKIPGSKVLFGGEPLENHS
Trypsin        SSSGLLDK--------KLEDLTIGPVLTVTTSSMIEHMK---------VLFGGEPLENHS

Recomb OsP5CDH IPEIYGAFKPTAVFVPLSEILKSGNFELVTREIFGPFQVVTEYSDDELELVLEACERMNA 480
Pepsin         IPEIYGAFKPTAVFVPLSEILKSGNFELVTREIFGPFQVVTEYSDDELE-----------
Trypsin        IPEIYGAFKPTAVFVPLSEILKSGNFELVTREIFGPFQVVTEYSDDELELVLEACERMNA

Recomb OsP5CDH HLTAAVVSNDPLFLQEVLGRSVNGTTYAGIRARTTGAPQNHWFGPAGDPRGAGIGTPEAI 540
Pepsin         --TAAVVSNDPLFLQEVLGRSVNGTTYAGIRARTTGAPQNHWFGPAGDPRGAGIGTPEAI
Trypsin        HLTAAVVSNDPLFLQEVLGRSVNGTTYAGIR--TTGAPQNHWFGPAGDPRGAGIGTPEAI

Recomb OsP5CDH KLVWSCHREIIYDIGPLPKNRALPSAT 568
Pepsin         KLVWSCHREIIYDIGPLPKNRALPSAT
Trypsin        KLVWSCHREIIYDIGPLPK--------
```

Supplementary Figure 1. Nucleotide and amino acid sequences of native and recombinant *Oryza sativa* P5C dehydrogenase. **A**: Alignment of the coding sequence of the constructs for expression of rice P5C dehydrogenase in *E. coli* with the coding sequence of the cDNA clone *J033091O16*. Sequences of the primers used for cloning are underlined. **B**: In the recombinant, truncated protein, the predicted mitochondrial transit peptide is replaced by 33 extra amino acids, but 27 of them are removed by TEV protease; the remaining residues upstream of start of the native protein are double underlined. Following SDS-PAGE, the protein was excised, destained, digested with either trypsin or pepsin and analyzed by LC-MS/MS using an LTQ-Orbitrap mass spectrometer (Thermo Fisher) and an Eksigent nano-HPLC. The dimensions of the reversed-phase LC column were 5 µm, 100 Å pore size C18 resin in a 75 µm i.d. × 10 cm long piece of fused silica capillary (Acclaim PepMap100, Thermo Scientific). After sample injection, the column was washed for 5 min with 95% mobile phase A (0.1% formic acid) and 5% mobile phase B (0.1% formic acid in acetonitrile), and peptides were eluted using a linear gradient of 5% mobile phase B to 40% mobile phase B in 65 min, then to 80% B for additional 5 min, at 250 nL/min. The LTQ-Orbitrap mass spectrometer was operated in a data dependent mode in which each full MS scan (30 000 resolving power) was followed by five MS/MS scans where the five most abundant molecular ions were dynamically selected and fragmented by collision-induced dissociation (CID) using a normalized collision energy of 35% in the LTQ ion trap. Dynamic exclusion was allowed. Tandem mass spectra were searched against predicted tryptic and petic peptides derived from the expected sequence with static cysteine alkylation by iodoacetamide and variable methionine oxidation using Mascot (Matrix Science).
